# Robust Identification of Alzheimer’s Disease subtypes based on cortical atrophy patterns

**DOI:** 10.1038/srep43270

**Published:** 2017-03-09

**Authors:** Jong-Yun Park, Han Kyu Na, Sungsoo Kim, Hyunwook Kim, Hee Jin Kim, Sang Won Seo, Duk L. Na, Cheol E. Han, Joon-Kyung Seong, Alzheimer’s Disease Neuroimaging Initiative, Michael Weiner, Paul Aisen, Ronald Petersen, Clifford R. Jack, William Jagust, John Q. Trojanowki, Arthur W. Toga, Laurel Beckett, Robert C. Green, Andrew J. Saykin, John Morris, Leslie M. Shaw, Enchi Liu, Tom Montine, Ronald G. Thomas, Michael Donohue, Sarah Walter, Devon Gessert, Tamie Sather, Gus Jiminez, Danielle Harvey, Matthew Bernstein, Nick Fox, Paul Thompson, Norbert Schuff, Charles DeCarli, Bret Borowski, Jeff Gunter, Matt Senjem, Prashanthi Vemuri, David Jones, Kejal Kantarci, Chad Ward, Robert A. Koeppe, Norm Foster, Eric M. Reiman, Kewei Chen, Chet Mathis, Susan Landau, Nigel J. Cairns, Erin Householder, Lisa Taylor Reinwald, Virginia Lee, Magdalena Korecka, Michal Figurski, Karen Crawford, Scott Neu, Tatiana M. Foroud, Steven G. Potkin, Li Shen, Faber Kelley, Sungeun Kim, Kwangsik Nho, Zaven Kachaturian, Richard Frank, Peter J. Snyder, Susan Molchan, Jeffrey Kaye, Joseph Quinn, Betty Lind, Raina Carter, Sara Dolen, Lon S. Schneider, Sonia Pawluczyk, Mauricio Beccera, Liberty Teodoro, Bryan M. Spann, James Brewer, Helen Vanderswag, Adam Fleisher, Judith L. Heidebrink, Joanne L. Lord, Sara S. Mason, Colleen S. Albers, David Knopman, Kris Johnson, Rachelle S. Doody, Javier Villanueva Meyer, Munir Chowdhury, Susan Rountree, Mimi Dang, Yaakov Stern, Lawrence S. Honig, Karen L. Bell, Beau Ances, Maria Carroll, Sue Leon, Mark A. Mintun, Stacy Schneider, Angela Oliver, Daniel Marson, Randall Griffith, David Clark, David Geldmacher, John Brockington, Erik Roberson, Hillel Grossman, Effie Mitsis, Leyla de Toledo-Morrell, Raj C. Shah, Ranjan Duara, Daniel Varon, Maria T. Greig, Peggy Roberts, Marilyn Albert, Chiadi Onyike, Daniel D’Agostino II, Stephanie Kielb, James E. Galvin, Dana M. Pogorelec, Brittany Cerbone, Christina A. Michel, Henry Rusinek, Mony J. de Leon, Lidia Glodzik, Susan De Santi, P. Murali Doraiswamy, Jeffrey R. Petrella, Terence Z. Wong, Steven E. Arnold, Jason H. Karlawish, David Wolk, Charles D. Smith, Greg Jicha, Peter Hardy, Partha Sinha, Elizabeth Oates, Gary Conrad, Oscar L. Lopez, MaryAnn Oakley, Donna M. Simpson, Anton P. Porsteinsson, Bonnie S. Goldstein, Kim Martin, Kelly M. Makino, M. Saleem Ismail, Connie Brand, Ruth A. Mulnard, Gaby Thai, Catherine Mc Adams Ortiz, Kyle Womack, Dana Mathews, Mary Quiceno, Ramon Diaz Arrastia, Richard King, Myron Weiner, Kristen Martin Cook, Michael DeVous, Allan I. Levey, James J. Lah, Janet S. Cellar, Jeffrey M. Burns, Heather S. Anderson, Russell H. Swerdlow, Liana Apostolova, Kathleen Tingus, Ellen Woo, Daniel H. S. Silverman, Po H. Lu, George Bartzokis, Neill R. Graff Radford, Francine Parfitt, Tracy Kendall, Heather Johnson, Martin R. Farlow, Ann Marie Hake, Brandy R. Matthews, Scott Herring, Cynthia Hunt, Christopher H. van Dyck, Richard E. Carson, Martha G. MacAvoy, Howard Chertkow, Howard Bergman, Chris Hosein, Sandra Black, Bojana Stefanovic, Curtis Caldwell, Ging Yuek Robin Hsiung, Howard Feldman, Benita Mudge, Michele Assaly, Dick Trost, Charles Bernick, Donna Munic, Diana Kerwin, Marek Marsel Mesulam, Kristine Lipowski, Chuang Kuo Wu, Nancy Johnson, Carl Sadowsky, Walter Martinez, Teresa Villena, Raymond Scott Turner, Kathleen Johnson, Brigid Reynolds, Reisa A. Sperling, Keith A. Johnson, Gad Marshall, Meghan Frey, Jerome Yesavage, Joy L. Taylor, Barton Lane, Allyson Rosen, Jared Tinklenberg, Marwan N. Sabbagh, Christine M. Belden, Sandra A. Jacobson, Sherye A. Sirrel, Neil Kowall, Ronald Killiany, Andrew E. Budson, Alexander Norbash, Patricia Lynn Johnson, Thomas O. Obisesan, Saba Wolday, Joanne Allard, Alan Lerner, Paula Ogrocki, Leon Hudson, Evan Fletcher, Owen Carmichael, John Olichney, Smita Kittur, Michael Borrie, T. Y. Lee, Rob Bartha, Sterling Johnson, Sanjay Asthana, Cynthia M. Carlsson, Adrian Preda, Dana Nguyen, Pierre Tariot, Stephanie Reeder, Vernice Bates, Horacio Capote, Michelle Rainka, Douglas W. Scharre, Maria Kataki, Anahita Adeli, Earl A. Zimmerman, Dzintra Celmins, Alice D. Brown, Godfrey D. Pearlson, Karen Blank, Karen Anderson, Robert B. Santulli, Tamar J. Kitzmiller, Eben S. Schwartz, Kaycee M. Sink, Jeff D. Williamson, Pradeep Garg, Franklin Watkins, Brian R. Ott, Henry Querfurth, Geoffrey Tremont, Stephen Salloway, Paul Malloy, Stephen Correia, Howard J. Rosen, Bruce L. Miller, Jacobo Mintzer, Kenneth Spicer, David Bachman, Elizabether Finger, Stephen Pasternak, Irina Rachinsky, John Rogers, Andrew Kertesz, Nunzio Pomara, Raymundo Hernando, Antero Sarrael, Susan K. Schultz, Laura L. Boles Ponto, Hyungsub Shim, Karen Elizabeth Smith, Norman Relkin, Gloria Chaing, Lisa Raudin, Amanda Smith, Kristin Fargher, Balebail Ashok Raj

**Affiliations:** 1 School of Biomedical Engineering, Korea University, Seoul, Republic of Korea; 2 Yonsei University College of Medicine, Seoul, Republic of Korea; 3 Department of Neurology, Sungkyunkwan University of Medicine, Seoul, Republic of Korea; 4 Department of Neurology, Samsung Medical Center, Seoul, Republic of Korea; 5 Department of Electronics and Information Engineering, Korea University, Sejong, Republic of Korea; 6 UC San Francisco, San Francisco, USA; 7 UC San Diego, La Jolla, USA; 8 Mayo Clinic, Rochester, MN USA; 9 UC Berkeley, Berkeley, San Francisco, USA; 10 University of Pennsylvania, Philadelphia, PA USA; 11 USC School of Medicine, Los Angeles, CA, USA; 12 University of California, Davis Sacramento, Sacramento, CA, USA; 13 Brigham and Women’s Hospital, Boston, MA, USA; 14 Indiana University, Bloomington, IN USA; 15 Washington University St. Louis, MO USA; 16 Janssen Alzheimer Immunotherapy, San Francisco, USA; 17 University of Washington, Seattle, WA, USA; 18 University of London, London, UK; 19 University of Michigan, Ann Arbor, MI, USA; 20 University of Utah, Salt Lake City, UT, USA; 21 Banner Alzheimer’s Institute, Phoenix, AZ, USA; 22 University of Pittsburgh, Pittsburgh, PA, USA; 23 University of Pennsylvania School of Medicine, Philadelphia, PA USA; 24 University of California Irvine, Irvine, CA, USA; 25 Khachaturian, Radebaugh & Associates, Inc, USA; 26 Ronald and Nancy Reagan’s Research Institute, Chicago, IL, USA; 27 General Electric, USA; 28 Brown University, Providence, RI. USA; 29 National Institute on Aging, Baltimore, Maryland, USA; 30 National Institutes of Health, USA; 31 Oregon Health and Science University, Portland, OR USA; 32 University of Southern California, Los Angeles, CA, USA; 33 Baylor College of Medicine, Houston, TX, USA; 34 Columbia University Medical Center, New York, NY, USA; 35 University of Alabama Birmingham, Birmingham, AL USA; 36 Mount Sinai School of Medicine, New York, NY, USA; 37 Rush University Medical Center, Chicago, IL, USA; 38 Wien Center, Miami Beach, FL, USA; 39 Johns Hopkins University, Baltimore, MD, USA; 40 New York University, New York, NY, USA; 41 Duke University Medical Center, Durham, NC, USA; 42 University of Kentucky, Lexington, KY, USA; 43 University of Rochester Medical Center, Rochester, NY, USA; 44 University of Texas Southwestern Medical School, Dallas, TX, USA; 45 Emory University, Atlanta, GA, USA; 46 University of Kansas, Medical Center, Kansas City, KS, USA; 47 Mayo Clinic, Jacksonville, Florida, USA; 48 Yale University School of Medicine, New Haven, CT, USA; 49 McGill Univ., Montreal Jewish General Hospital, Montreal, QC, Canada; 50 Sunnybrook Health Sciences, Toronto, ON, Canada; 51 U.B.C. Clinic for AD & Related Disorders, Vancouver, BC, Canada; 52 Cognitive Neurology St. Joseph’s, Ontario, ON, Canada; 53 Cleveland Clinic Lou Ruvo Center for Brain Health, Las Vegas, NV, USA; 54 Northwestern University, Evanston, IL, USA; 55 Premiere Research Inst, West Palm Beach, FL, USA; 56 Georgetown University Medical Center, Washington, DC, USA; 57 Stanford University, Stanford, CA, USA; 58 Banner Sun Health Research Institute, Sun City, AZ, USA; 59 Boston University, Boston, MA, USA; 60 Howard University, Washington, DC, USA; 61 Case Western Reserve University, Cleveland, OH, USA; 62 Neurological Care of CNY, Liverpool, NY, USA; 63 Parkwood Hospital, London, ON, Canada; 64 University of Wisconsin, Madison, WI, USA; 65 Dent Neurologic Institute, Amherst, NY, USA; 66 Ohio State University, Columbus, OH, USA; 67 Albany Medical College, Albany, NY, USA; 68 Hartford Hosp, Olin Neuropsychiatry Research Center, Hartford, CT, USA; 69 Dartmouth Hitchcock Medical Center, Lebanon, NH, USA; 70 Wake Forest University Health Sciences, Winston-Salem, NC, USA; 71 Rhode Island Hospital, Providence, RI, USA; 72 Butler Hospital, Providence, RI, USA; 73 Medical University South Carolina, Charleston, SC, USA; 74 St. Joseph’s Health Care, Irvine, CA, USA; 75 Nathan Kline Institute, Orangeburg, NY, USA; 76 University of Iowa College of Medicine, Iowa City, Iowa City, IA, USA; 77 Cornell University, Ithaca, NY, USA; 78 University of South Florida: USF Health Byrd Alzheimer’s Institute, Tampa, FL, USA

## Abstract

Accumulating evidence suggests that Alzheimer’s disease (AD) is heterogenous and can be classified into several subtypes. Here, we propose a robust subtyping method for AD based on cortical atrophy patterns and graph theory. We calculated similarities between subjects in their atrophy patterns throughout the whole brain, and clustered subjects with similar atrophy patterns using the Louvain method for modular organization extraction. We applied our method to AD patients recruited at Samsung Medical Center and externally validated our method by using the AD Neuroimaging Initiative (*ADNI*) dataset. Our method categorized very mild AD into three clinically distinct subtypes with high reproducibility (>90%); the parietal-predominant (P), medial temporal-predominant (MT), and diffuse (D) atrophy subtype. The P subtype showed the worst clinical presentation throughout the cognitive domains, while the MT and D subtypes exhibited relatively mild presentation. The MT subtype revealed more impaired language and executive function compared to the D subtype.

Alzheimer’s disease (AD) is a neurodegenerative disorder characterized by deficits in multiple cognitive domains, and worsens across an increasingly broader range of domains as the disease progresses^1^. There remains a wide spectrum of clinical features in AD patients, ranging from atypical cognitive dysfunction at presentation (starting with language, visuospatial or frontal executive dysfunction rather than memory impairment) to different rates of disease progression^1^,^2^,^3^. The existence of the aforementioned distinct clinical phenotypes among patients supports the hypothesis that AD consists of several subtypes. The identification of such subtypes may potentially improve our understanding of the underlying pathomechanisms of the disease, prediction of disease course, and the development of new disease-modifying treatments^4^. A recent post-mortem neuropathological study has suggested the existence of three distinct subtypes, based on the distribution of neurofibrillary tangles^5^. However, this post-mortem subtyping approach is limited to AD patients in their advanced stages and autopsies cannot map the entire human brain.

Recent advances in neuroimaging have greatly improved AD subtyping attempts. A computational approach to subtyping has been suggested for AD patients via hierarchical clustering analysis^6^,^7^,^8^, which computes the similarity between any pair of subjects in terms of cortical thickness of the whole brain^6^,^7^, or a few selected neuroimaging measures^8^, and then aggregates subjects in order of descending similarity. With an arbitrary threshold of aggregation levels, this produces plausible AD subtypes. However, this approach is vulnerable to the sampling bias of a used dataset, therefore generating different subtypes even with slight changes in the sampled dataset, and the outcomes tend to cluster based on overall similarity of the cortical thickness rather than cortical atrophy patterns, as it utilizes a summation of pairwise differences.

Here we present a novel method for AD subtyping utilizing graph theory, which has high reproducibility against to a random perturbation in the sampled dataset. We calculated the similarity between any two subjects in their cortical atrophy patterns across the whole brain, and clustered subjects with similar cortical atrophy patterns using the Louvain method, which was developed for modular organization extraction^9^. While hierarchical clustering identifies subtypes deterministically, the Louvain method explores modular organization stochastically and is thus more robust against sampling bias.

We hypothesized that this approach would identify subtypes with distinct atrophy patterns and cognitive profiles, and applied the method to patients with ‘very mild AD’^10^,^11^, and compared the clinical manifestations of the resultant AD subtypes using two different datasets: the Samsung Medical Center (SMC) dataset consisting of 225 patients with AD, and 320 age, gender and education level-matched CN subjects, and the external validation dataset from the Alzheimer’s Disease Neuroimaging Initiative (ADNI validation dataset) consisting of 131 AD patients and 158 matched CN subjects (see Table 1 and Supplementary Table S1 for details). The rationale for selecting very mild AD patients is because predictive markers of disease progression would be potentially more useful in patients with earlier stages, and as the disease progresses, their atrophy becomes widespread resulting in less distinctive atrophy patterns. Moreover, we limited the study to patients with minimal white matter hyperintensities in order to exclude patients with mixed Alzheimer and vascular pathology that may affect the cortical thickness.

In this present study, we established a novel subtyping approach that categorizes the early stage AD into several subtypes based on the pattern of cortical atrophy. By applying this approach to patients with very mild AD, we identified three anatomical subtypes with distinct neuropsychological profiles which were strongly associated with their characteristic atrophy patterns. The method showed high reproducibility and was also externally validated by the ADNI validation dataset.

## Results

### AD subtyping based on distinct cortical atrophy patterns

**Figure 1:**
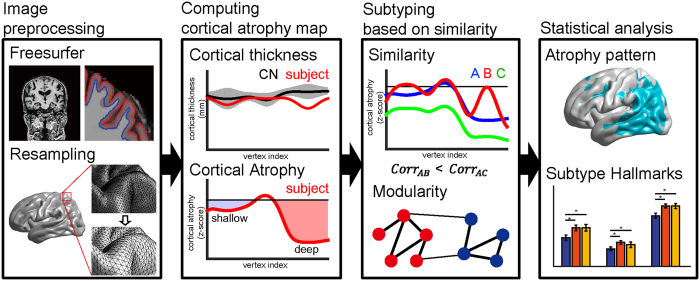
Overview of the proposed method. After brain surface information is extracted, resampling is performed and noise is removed. Z-scores are then computed for each subject’s cortical thickness with respect to the cognitively normal (CN) subjects as their individual ‘cortical atrophy’. The similarity of any pair of subjects is defined using a correlation coefficient between cortical atrophy levels of the subjects. Similarity is therefore more sensitive to the shape of the cortical atrophy patterns, rather than overall levels (*corr*_*AB*_ > *corr*_*AC*_). Modular organization of subjects was extracted using the defined similarity. Note: cortical atrophy plots in the second and third boxes of the overall pipeline are depicted as an example for illustration purposes.

**Figure 2:**
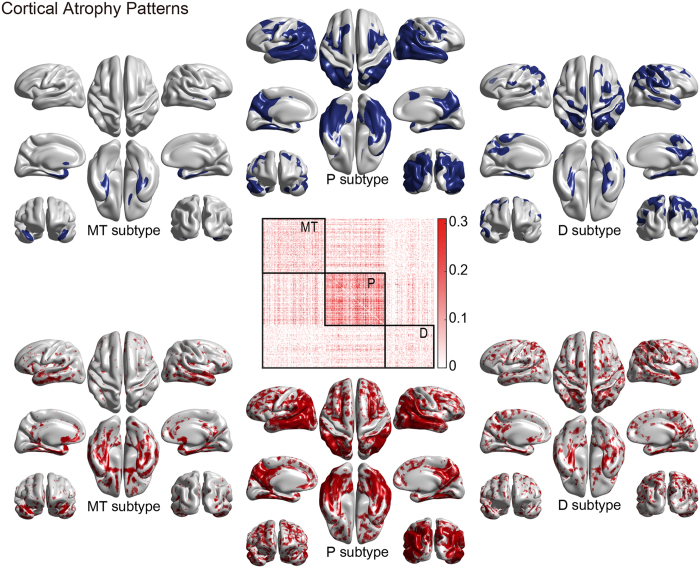
Cortical atrophy patterns for three AD subtypes using the SMC dataset: MT (medial temporal-predominant), P (parietal-predominant), and D (diffuse) subtypes. Modular organization of the subjects was achieved using defined similarity and reordered to illustrate subtyping where each square captures a subtype border. Group comparison results of cortical thicknesses between each subtype and CN was corrected using random field theory and regions with corrected *p* < 0.001 are visualized (p < 0.05 for the D subtype) with covariate age, gender and education. (upper row). Atrophy map shows medians of the cortical atrophy (z-scores) in each subtype (−0.6 ≤ z ≤ −0.3) (lower row).

We identified AD subtypes by clustering AD patients based on the similarity of cortical atrophy patterns: if a subset of subjects shared a similar cortical atrophy pattern, we grouped them together. The overview of this procedure is summarized in Fig. 1. First, we computed the cortical atrophy pattern of each AD patient using normalized cortical thickness data. We then constructed a similarity matrix based on correlation coefficients of the cortical atrophy patterns for any two AD patients. Finally, clusters of AD patients with similar cortical atrophy patterns were detected using the Louvain method^9^ which is the state-of-the-art modular organization extraction method in network science. The modular organization can be found by maximizing a value of modularity that is high when the intra-modular connections are dense while the inter-modular connections are sparse (Fig. 2 and Supplementary Figure S1 middle). This subtyping produced statistically significant results (permutation testing for similarity matrix, p < 0.001, see Supplementary materials).

The subtypes were named after their statistically significant characteristic atrophy patterns found in the atrophy pattern comparison to CN (Fig. 2 and Supplementary Figure S1 upper row)^6^,^7^. In the SMC dataset, we obtained three subtypes: an MT subtype (medial temporal-predominant atrophy, *n* = 82), P subtype (parietal-predominant atrophy, *n* = 79), and D subtype (diffuse atrophy, *n* = 64). The MT subtype features major atrophy of the medial temporal lobe including the entorhinal cortices of both hemispheres. In contrast, the P subtype’s major atrophy is of the parietal cortices, superior and lateral temporal lobes and precuneus of both hemispheres. Although the atrophy extended into the frontal lobes, the depth of atrophy was greater than in the parietal lobes and precuneus. The D subtype featured sporadic atrophy over the cortices; however, the atrophy map showed that it was shallow and diffused (spanned) over the cortices, in contrast to the focal atrophy of the other two subtypes. The cortical atophy patterns of each subtype in the ADNI validation dataset showed the same trend with those of the SMC dataset.

### Neuropsychological test results

Our subtypes solely determined by MR image analysis exhibited distinct neuropsychological characteristics across the three subtypes (Table 2 and Supplementary Table S2). Overall, the P subtype showed the worst performance in overall cognitive domains, while cognitive function in the MT and D subtypes was relatively spared. The MT and D subtypes presented similar cognitive profiles except for language function, which was more disrupted in the MT subtype. Specifically, in the SMC dataset (Table 2), the P subtype consisted of patients with the youngest age and highest education level compared to the other two subtypes. However, they revealed the worst cognitive profiles in terms of attention, visuospatial, visual memory, and frontal executive function. Above all, the attention and frontal executive function of the P subtype was severely impaired as determined by the worst scores in digit-span backward (p = 0.004), COWAT semantic fluency (animal [p = 0.002] and supermarket items [p < 0.001]), and the Stroop test (p < 0.001). In the RCFT copy test, not only was the score lower (p < 0.001) but also the time required to complete the task was longer for the P subtype patients (p < 0.001), exposing a significant deficit in visuospatial function. In line with this, the most severe impairment in visual memory was seen in the P subtype, as revealed by the immediate and delayed recall task in RCFT. Verbal memory function did not differ across the three subtypes in SVLT delayed recall (p = 0.349). When it comes to parietal lobe specific function, the dysfunction in calculation (p = 0.053) and ideomotor praxis (p = 0.004) was the worst in the P subtype patients. Even after stratifying the AD patient according to their age of onset, the P subtype exhibited the worst cognitive function in both early onset AD (EOAD, onset age < 65 years) and late onset AD (LOAD, onset age ≥ 65 years) (Supplementary Table S3).

In comparison, the MT and D subtypes exhibited relatively benign impairment. In terms of language domains, the D subtype showed relatively mild impairment in comparison to the other two subtypes (MT vs D, p = 0.008; P vs D, p = 0.026) in the K-BNT. In contrast, the MT subtype scored the worst in the K-BNT, although the impairment was not significantly lower than the P subtype (p = 0.659). In the Animal Category Fluency Test, the MT subtype patients showed worse performance when compared to D subtype patients (p = 0.077), though it did not reach statistical significance. Also the ADNI validation dataset have similar clinical characteristics across the subtypes.

### Cortical atrophy hallmarks of each subtype

**Figure 3:**
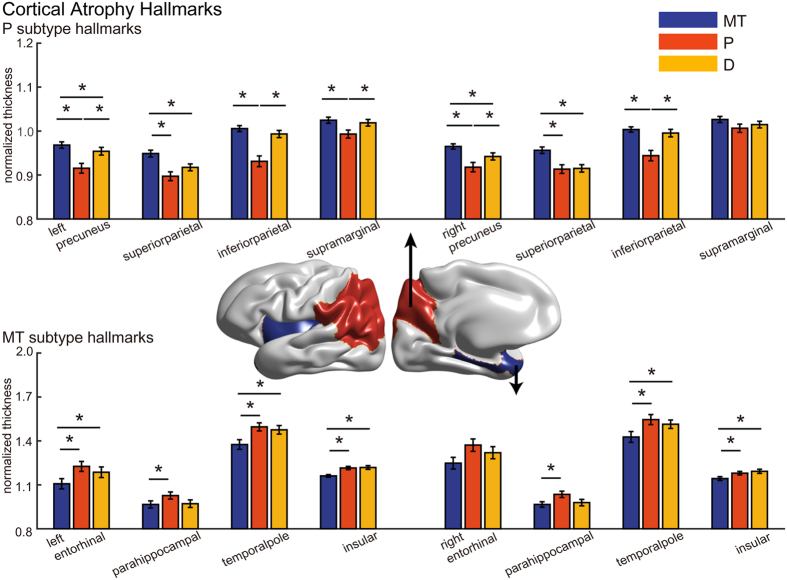
Cortical atrophy hallmarks in each AD subtype in the SMC dataset. Normalized cortical thicknesses of the subtype-specific hallmark regions are shown: P subtype hallmarks (upper right), MT subtype hallmarks (lower right) and D subtype hallmarks (left). Bar colors represent specific subtypes: blue (MT subtype), red (P subtype) and yellow (D subtype), where asterisks indicate statistical significance (permutation-based ANCOVA, FDR-adjusted).

Each subtype revealed its own distinct cortical atrophy patterns. We therefore selected the characteristic regions of each subtype that showed severe atrophy in both the SMC dataset and the ADNI validation dataset through ROI-based analysis, and defined them as the ‘hallmarks’ of cortical atrophy for each subtype. The MT subtype showed characteristic atrophy patterns in the entorhinal cortex, the parahippocampal cortex, the temporal pole and the insular cortex (FDR-adjusted; Fig. 3 and Supplementary Figure S2, the lower panels), while the P subtype showed significant thinning in the precuneus and regions in the parietal lobe (the supramarginal and the inferior and superior parietal cortices, FDR-adjusted; Fig. 3 and Supplementary Figure S2, the upper panels). When it comes to the subcortical structures, the volume of the hippocampus and amygdala was smaller in the MT subtype (Supplementary Table S4). Compared to the other two subtypes, the atrophied region in the D subtype was relatively unclear and sometimes failed to show convergent findings in both datasets. In general, hallmarks in the left and the right hemispheres showed a similar trend. The summary of the hallmark analysis results can be found in Supplementary Table S5. We note larger atrophy levels in the D subtype in the ADNI validation dataset compared to those in the SMC dataset (Fig. 2 and Supplementary Figure S1).

### Reproducibility of subtyping

Our proposed method is highly reproducible (SMC dataset: 92.25%; ADNI validation dataset: 92.53%) and consistent on average for 10 subsets with 10% random removal. We extensively compared the proposed method with the following two methods: hierarchical clustering (HC) and the Louvain method based on the correlation and Euclidean distance between subjects. Our proposed method with correlation coefficients between cortical atrophy patterns excelled HC since it had the highest modularity and high reproducibility (92.25% reproducibility, Table 3). The hierarchical clustering (HC) method has lower reproducibility than our method (83.42% reproducibility, Table 3). Since HC deterministically clusters subjects, the subtyping results can be biased to the sample distribution while the Louvain method has increased chance to find optimal subtyping and leading to the higher reproducibility. Our extensive analysis (Supplementary Tables S6 and S7) also showed that using correlation coefficients lead better clinical association.

## Discussion

In this paper, we proposed a new subtyping approach that uses similarity in cortical atrophy patterns based on correlation coefficients and the Louvain method for clustering subjects. The proposed method successfully categorizes very mild AD into clinically distinct anatomical subtypes with high reproducibility.

Our study holds several methodological strengths over previous approaches^6^. First, our method provides highly reproducible subtypes (>90%), as tested in two different ethnic populations and subsets of samples with 10% random removal. Moreover, despite the strength of the magnetic field of MR scanners are different with each other in two datasets (the SMC dataset: 3T, the ADNI validation dataset: 1.5T), the subtyping results were fairly reproduced. Second, our strategy is based on cortical atrophy patterns rather than raw cortical thickness and therefore subjects within a single subtype naturally share similar cortical atrophy patterns. Third, we employ the Louvain method for clustering, a tool known to be accurate and efficient. Fourth, there is no heterogeneous subtype that consists of the ‘leftovers’ or those cases that cannot be classified into a distinct subtype. In this regard, our approach addresses the limitations of a previous study that classifies such cases as one subtype, which may be a source of bias^5^. Lastly, we externally validated our methods by applying our subtyping strategy to the ADNI validation dataset and achieved similar results to those of the SMC dataset.

The proposed subtypes have distinct neuropsychological characteristics in the SMC dataset (Table 2). Patients in the P subtype had an earlier onset of disease and exhibited the worst clinical outcomes among the three subtypes in most cognitive domains, except for verbal memory function. These findings may be explained by the regions involved in cortical atrophy in the P subtype: the precuneus, bilateral posterior parietal cortices (inferior and superior parietal lobules), and bilateral dorsolateral frontal areas. The most representative function of the parietal cortex is spatial processing^12^,^13^,^14^, and deficit in visuospatial function and visual memory was accordingly evident in the P subtype. Besides visuospatial function, the parietal lobe (both lateral parietal cortices and the precuneus) is also involved in selective attention and working memory^15^, and therefore correlates well with overall deterioration in the P subtype. In particular, the precneus, in which atrophy was most evident in the P subtype, plays a key role in a wide range of higher-order cognitive functions, such as executive function, visuospatial imagery, and self-processing operations^13^. In addition, the dysfunction in calculation (p = 0.053) and ideomotor praxis (p = 0.004), which are regarded as parietal specific function^16^,^17^,^18^, was the worst in the P subtype patients.

In comparison to the P subtype patients, the MT and D subtype patients showed better neuropsychological performances and their overall cognitive profile were quite similar. However, even in these two relatively mild anatomical subtypes, MT subtype patients showed worse performances in language domain (K-BNT), processing speed in visuospatial constructive function (RCFT copy time) compared with the D subtype patients. Moreover, MT and D subtypes differed in the average age, gender distribution and the pattern of gray matter atrophy, implying that MT and D subtype may be a distinct subtype. However, there lies a possibility that MT and D subtype may be quite difficult to distinguish mainly based on their neuropsychological performances. This also implies that differentiation of AD subtypes (based on the proposed cortical atrophy pattrerns) may be helpful when enrolling participants and analyzing data in future clinical trials. If a single group consists of both MT and D subtypes, the response to treatments or underlying pathophysiology might be distinctly different, and the outcomes may be affected accordingly.

Some may argue that the aforementioned distinct cognitive profile may be a result of generally worse cognition or stage of disease progression rather than reflecting a specific clinical subtype. In order to avoid this issue, we introduced the concept of correlation coefficients to subtype AD patients based on the similarities in the overall cortical thinning patterns (predominant atrophy pattern) rather than based merely on the absolute values of cortical thickness. Moreover, we included the patients with CDR-SOB ≤4 (i.e., very mild AD) to narrow the stage of disease progression so as to avoid classification of AD into subtypes with different stage of disease. In this present study, the disease duration, the MMSE, and CDR-SOB scores which are known to reflect the general cogntive status and stage of disease did not show significant difference across the subtypes. Consequently, our proposed subtypes appear to reflect specific clinical subtype rather than a result of AD with different stage of disease.

Of note, the P subtype shares several common core features with the hippocampal-sparing subtype, a neuropathologically defined subtype characterized by aggressive disease progression, earlier age of onset, and cortical atrophy involving the parieto-frontal cortices^5^,^19^. In addition, there also exists similarities between our MT subtype and the limbic-predominant AD in terms of female predominance, relatively late age of disease onset, and the atrophy pattern restricted to the medial temporal lobe^5^,^19^. Intriguingly, the MT subtype showed more atrophied hippocampus and amygdala compared to the P subtype (Supplementary Table S4), which was also a core finding in the limbic-predominant and hippocampal-sparing AD^19^. This substantial overlap between neuropathology studies and ours lend greater weight to the subtypes identified in this present study.

Despite numerous attempts to classify AD into multiple subtypes, there have been few effective measures proposed to accomplish the task^6^,^8^,^20^,^21^. Some of the recent studies exploited gray matter volume of a few selected regions of interest^8^ or the whole brain^21^. The study called CHIMERA^21^ proposed a notable probabilistic subtyping framework using the volume of 80 regions of interest. The proposed probabilistic method is promising but its main limitation is that it is not suitable for the high-dimensional data like our whole brain cortical thickness data. Murray *et al*. proposed pathological AD subtypes by sorting along an axis that represents the ratio of hippocampal to cortical neurofibrillary tangle density in a clinicopathological cohort of 889 cases of AD^5^,^22^. Although this study provided AD subtypes with a relatively strong level of evidence, it was limited as the subtyping was performed post-mortem, representing AD subtypes only in their advanced stages. Although, the distinct cortical atrophy patterns across the pathological subtypes was also investigated in a follow-up neuroimaging study by utilizing the first MRI after diagnosis of AD^19^, there still remains a limitation in that the subtyping itself was performed based on post-mortem tauopathy. This approach also cannot be used to determine which subtype an early stage AD patient should be assigned to, and autopsies cannot map the entire brain as it is a region-of-interest–based method. In contrast to the post-mortem study, subtyping using MR imaging is non-invasive and thus applicable to living patients. Our findings also provide evidence that subtyping can be achieved for very early stage AD, and thus may be useful for early treatment intervention.

Our proposed method is highly reproducible, showing an average consistency of 92.25% and 92.53% for 10 subsets with 10% random removal in the SMC dataset and ADNI validation dataset, respectively. Our analysis (Table 3) revealed that the Louvain method for modular organization extraction improves the reproducibility, showing higher reproducibility compared to the hierarchical clustering method (SMC dataset, 83.42%; ADNI validation dataset, 72.03%). Since the hierarchical clustering method clusters subjects deterministically, it tends to be vulnerable to changes in sample size or distribution. In contrast, the Louvain method stochastically explores the optimal clustering configuration in order to maximize the modularity value, thereby increasing the chance to identify optimal subtyping and thus leading to the higher reproducibility.

Our analysis (Table 3) showed that the subtyping strategy utilizing the correlation coefficients tends to classify AD into distinctive subtypes better than approaches based on Euclidean distance. This superiority arises from the nature of the correlation coefficient itself in that the absolute values of cortical thickness can be automatically controlled in the operation process and thus using correlation coefficients is useful to capture similarities in cortical thinning patterns between the two subjects, rather than the differences in thickness, and is therefore more sensitive in distinguishing the pattern difference (Fig. 1). We emphasize that our D subtype is neither a leftover group (i.e. not classifiable into MT or P subtypes) nor a group defined by the level of cortical thinning. Since the proposed method clusters all subjects simultaneously based on the similarity of the cortical atrophy patterns, patients with the D subtype share their own distinct atrophy pattern that is distinguishable from the other subtypes. Also since we employed the correlation coefficients as a similarity metric, the overall cortical thinning was not a factor for clustering, while the Euclidean distance-based measure could be affected by the sum of differences in the cortical thickness. Thus, we further compared our method with the Euclidean distance-based Louvain method. The Louvain method based on the Euclidean distance resulted in two subtypes; one with a low level of overall cortical thinning and the other with a high level, since the Euclidean distance-based method integrates overall atrophy of the cerebral cortex. Although the low-level atrophy subtype can be classified into the MT and P subtypes, the difference in cognitive profile was less clear than with our method (Supplementary Tables S6 and S7).

Another novelty in our method is using the z-score of cortical thickness normalized by the distribution of cortical thickness in the CN group, instead of using raw cortical thickness. The raw cortical thickness is a snapshot of the current remnants; however, for the cluster analysis of the cortical thinning pattern, it is better to measure the extent of cortical atrophy in each patient instead. Generally, it is not possible to observe the extent of cortical thinning within a single MR image, as we cannot determine the cortical thickness of the brain before AD diagnosis. In this study, we estimated the level of cortical atrophy by normalizing the remnant cortical thickness with the distribution of cortical thickness in the CN group. The standard deviation for each brain region may account for inter-subject variability; in cases where large inter-subject variability resides, even when the remnant is small, it would not be considered as severe cortical atrophy (Fig. 1). Since this procedure considers the inter-subject variability, where the large inter-subject variability lies, the resultant z-score is not low even when the subject’s cortical thickness is thinner than the mean of the CN subjects. Thus, in the resultant cortical atrophy patterns, the deep or shallow atrophy is determined not only by the cortical thinning of the patient, but also by the inter-subject variability in the CN group. We believe that the use of this z-score improves subtyping performance.

Although the heterogeneity of AD is not a new concept, the underlying mechanisms that can account for the selective vulnerability in topographic distribution of brain atrophy in each AD subtype remain elusive. The difference in tau pathology, functional networks, metabolism may play a major role in selective vulnerability^23^, but we cannot support any of the previous hypotheses with our data of this present study. In this regard, we at least aim to suggest the “hallmarks” of cortical atrophy for each subtype so that they may serve as a viable start point in future studies. In the MT subtype, the entorhinal cortex, the parahippocampal cortex, the temporal pole and the insular cortex were selected through ROI-based analysis as hallmarks. Atrophy in these structures are known to produce selective vulnerability presented in AD patients, especially responsible memory dysfunction^24^. Moreover, temporal pole atrophy is known to play a prominent role in naming impairment, which was also evident in our study^25^. In the P subtype, the precuneus, the supramarginal, inferior parietal and superior parietal cortices were selected as hallmarks. Consistent with the damage in these structures, the P subtype showed significant impairment throughout the overall cognitive function, especially for visuospatial and executive function^12^,^13^,^14^,^15^. Of note, the D subtype revealed a balanced atrophy pattern rather than involving focal specific region. Although significant thinning near the central sulcus was observed in the D subtype, this finding might not be a result of selective vulnerability in the region. Rather, this may be a relative finding as the D subtype tends to reveal shallow but diffuse atrophy throughout the cortices, while paracentral cortices are less affected in the other subtypes. Though we cannot point out a selectively vulnerable region for this subtype, this balanced atrophy pattern itself may act as a hallmark for the D subtype.

The hallmarks may provide not only supportive evidence that subjects clustered by our approach share a specific atrophy pattern, but also informative criteria to distinguish subtypes in future. As an example, if a certain subject presents with severe alteration of cortical thickness in the precuneus and a few parietal regions, they may be suspected to be a P subtype patient, since the precuneus and a few parietal regions are hallmarks of the P subtype.

As neurodegenerative diseases are hypothesized to propagate along the brain network, the possible network involved in each subtype may be important in understanding the heterogeneity of AD. In light of the pattern of atrophy, the AD pathology of the P subtype appears to be centered preferentially along the default mode network. The parietal lobe is well-known to be responsible for higher cortical function in human and thereby associated with high metabolic demand, but at the same time, known as the most vulnerable region due to thin myelination^7^,^26^. When considering that the degree of parietal vulnerability is highly variable across individuals^26^,^27^, we surmise that patients who show such vulnerability may develop P subtype of AD dementia. By contrast, the anterior medial temporal network, a network involved in some aspects of declarative memory^28^, appears to be preferentially disrupted in the MT subtype when taking the memory and confrontation naming dysfunction into consideration. However, further connectivity studies are necessary in order to find the vulnerable networks of each subtype.

We note several limitations to our proposal. First, the regularization parameter, γ, for the Louvain method was set empirically, which may act as a potential bias. We followed the previous post-moterm neuropathological study^5^ for the target number of subtypes and controlled the parameter in order to obtain three subtypes. However, as shown in our experimental results, the proposed method provides consistent subtyping across different population sets with distinct cortical atrophy patterns and the associated neuropsychological tests scores were validated from a clinical perspective. Second, pathological confirmation was not performed in this study. We validated our subtyping approach using the ADNI validation dataset and provided supporting evidence using neuropsychological tests, inter-group consistency of subtypes, and distinct cortical regions of each subtype (hallmarks). Third, we cannot exclude the possibility that the cortical atrophy was affected by non-AD pathology. In order to minimize the contribution of factors related to cortical atrophy other than AD, we used probable AD patients with minimal WMH only and excluded those with moderate or severe WMH as well as patients with a past history that may contribute to cognitive deficit. Although this may limit the generalizability of the study, as nearly half of all AD patients tend to reveal moderate or severe WMH^29^,^30^,^31^, this aided in analyzing the cortical atrophy patterns affected mainly by AD itself. Fourth, our study cannot explain the mechanisms of selective vulnerability in each subtype. Instead, we listed up the core atrophied subregions of each subtype (hallmarks) in order to identify regions that physicians could focus on when designing future studies. Fifth, our study did not include the subcortical regions and other important biomarker including the amount of amyloid beta and tau protein deposition. For example, it is well-known that the volume of hippocampus and amygdala was associated with cognitive performance of dementia patients^32^. Though we did not include the subcortical regions, it may be acompanied with the structure nearby in the medial temporal lobe. Our study excluded the biomarkers beyond neuroimaging though they are important and reliable diagnostic biomarkers, since we believe that the neuroimaging biomarkers from the MR imaging is more appropriate for screening the population. Sixth, we cannot exclude the possibility that the distinct clinical characteristics across the subtypes might have been affected by the age of onset (i.e, EOAD and LOAD). However, it should be noted that the overall cognitive profiles of each subtypes were similar even after stratifying the subtypes according to age of disease onset, indicating that our findings are not simply a result of disproportional distribution of EOAD and LOAD patients across subtypes. Lastly, no longitudinal follow-up study was performed in this setting, limiting the potential value as we cannot determine whether the proposed subtypes can be used for the prediction of disease progression.

## Summary and Conclusion

We propose a novel subtyping method for very mild AD patients. The resulting subtypes are strongly associated with neuropsychological performance and each has a distinct cortical atrophy pattern. The suggested method has high reproducibility in comparison to previously suggested methods. However, it remains to be determined in future studies whether this subtyping approach can be applied at more advanced stages (mild, moderate, severe AD) or whether proposed subtypes in very mild AD may entail differing predictive values for progression trends in AD. Applying this subtyping approach to other diseases and diagnostic purposes may also yield further benefit.

## Materials and Methods

### Subject recruitment and MR image acquisition

In the SMC dataset, we retrospectively analyzed the data of 225 patients with AD and 320 age, gender and education level-matched cognitively normal subjects (CN) at Samsung Medical Center from June 2006 through December 2013. Written informed consent for the study was obtained from all patients and the protocol was approved by the Institutional Review Board of Samsung Medical Center. This study followed the tenets of the Declaration of Helsinki in 1964 and all subsequent revisions. We obtained high-resolution T1-weighted MR images using a 3.0T Philips Achieva.

In order to provide additional evidence that our findings are accurate and applicable to the general AD dementia population, we applied our subtyping approach to the Alzheimer’s Disease Neuroimaging Initiative (ADNI) database (adni.loni.usc.edu). ADNI was launched in 2003 as a public-private partnership, led by Principal Investigator Michael W. Weiner. The primary goal of ADNI has been to test whether serial MRI, positron emission tomography (PET), other biological markers, and clinical and neuropsychological assessment can be combined to measure the progression of mild cognitive impairment (MCI) and early AD (for updated information, see www.adni-info.org) (see Supplementary Figure S5 for details). A total of 131 patients with AD and 158 age, gender and education level-matched CN subjects were selected for the ADNI validation dataset. The T1-weighted MR images for the subjects were recorded following the ADNI acquisition protocol^33^. We used images acquired at 1.5T for the ADNI validation dataset. In both datasets, we included AD patients fulfilling the following criteria: clinical dementia rating sum-of-boxes (CDR-SB) less than or equal to 4 (very mild AD)^10^,^11^, MMSE score less than 27^34^,^35^, and minimal white matter hyperintensities (WMH)^30^,^31^. The detailed subject inclusion/exclusion criteria and MR protocol are described in the Supplementary material.

### Neuropsychological assessment

The cognitive function of each participant in the SMC dataset was assessed using a standardized neuropsychological assessment tool, the Seoul Neuropsychological Screening Battery (SNSB)^36^,^37^,^38^. The SNSB includes tests designed to measure attention, language, praxis, visuoconstructive function, verbal and visual memory, and frontal executive function^36^,^37^. We used standard neuropsychological test scores (z-scores) because the age, sex, and education levels were different between the AD dementia subtypes. The z-scores were derived based on age- and education-adjusted norms^36^,^37^. In addition to the SNSB, we also used the Korean version of the Mini-Mental Status Exam (K-MMSE) and CDR-SB. The ADNI neuropsychological assessment procedures have been previously described^39^,^40^. We used a modified Alzheimer’s Disease Assessment Scale - cognitive subscale (ADAS-cog)^41^,^42^, the most widely used standard cognitive measure in the AD population, and included the Digit Span Test, BNT^43^, Rey Auditory Verbal Learning Test (RAVLT)^44^, Clock Drawing Test^45^, Trail Making Test (TMT)^46^,^47^, Digit Symbol Substitution Test^48^ and Category Fluency Test^40^. Composite scores including ADNI-EF and ADNI-Mem were also used^49^,^50^.

### Image preprocessing

Figure 1 depicts a brief overview of the proposed analysis pipeline. We computed the cortical thickness from T1-weighted images using FreeSurfer v5.1.0^51^,^52^. Following the recommended reconstruction pipeline, we visually checked and corrected image segmentation. FreeSurfer extracts various surfaces from T1-weighted images, including the pial surfaces (outer boundary of the gray matter), white matter surfaces (boundary between gray and white matter), and spherically deformed surfaces which are registered to the FreeSurfer’s standard subject. Due to inter-subject variability of brain shapes, we resampled the surfaces with 40,962 vertices for each hemisphere using our in-house software^53^. We then removed artifacts in the cortical thickness data using the Laplace-Beltrami (LB) operator, similarly to past studies^53^,^54^,^55^.

### Estimation of cortical atrophy patterns using z-scores

For each vertex of a cortical surface, a z-score was computed with respect to the cortical thickness distribution in the CN group: 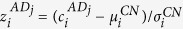, where 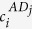 is the cortical thickness value of the *i*-th vertex of the *j*-th AD patient, 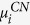 is the mean cortical thickness of the *i*-th vertex in the CN group, and 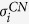 is its standard deviation. This z-score represents the extent of cortical atrophy for a specific location. Once the relative extent of cortical thinning is calculated for all vertices sampled over the smooth cortical surface, we then define a cortical atrophy pattern of the *j*-th AD patient by concatenating 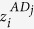, for *i* = 1, 2, …, 81924.

### Similarity matrix construction and subtyping using the Louvain method

We constructed a similarity matrix for the AD group using correlation coefficients between cortical atrophy patterns of any two subjects. We excluded the non-cortical tissue while computing the correlation coefficients. For cluster analysis of the AD patients, we employed the Louvain method^9^ which was developed for modular organization extraction in network science. The modular organization in a large network can be found by maximizing a value of modularity which is high when the intra-modular connections are dense while the inter-modular connections are sparse. In our problem setting, dense intra-modular connections imply high similarity of the cortical atrophy pattern between subjects in a module. The Louvain method is not only efficient for larger networks, but also very accurate; for a few large networks, its value of modularity was the highest among the current common modular organization extraction methods^9^. Since the Louvain method is based on a greedy optimization method, the clustering results can vary slightly. To resolve this issue, we employed a ‘major voting’ scheme, in which we extracted modular organization *N* times, and labeled a subject with the most frequently assigned cluster (Supplementary Figure S4). The Louvain method has a resolution parameter, γ (gamma), which controls the number of clusters. In our experiments, we controlled it to obtain three subtypes based on the previous post-mortem study^5^ (*N* = 1000 and γ = 0.9). We also estimated statistical significance of the Louvain method based on a permutation test of the similarity matrix (see Supplementary material). We used the implementation in the brain connectivity toolbox for the Louvain method^56^.

### Comparison of the proposed method with other methods

To compare with the previous study^6^, we followed the same method as possible as we can; the only difference is that we used our estimated cortical atrophy instead of cortical thickness for fair comparison and adopt both correlation coefficient and Euclidean distance. The latter was for investigating the effects of the correlation coefficients. Since the Louvain method requires similarity measures, we transformed the Euclidean distance into the similarity with monotonically decreasing function (*w*_*ij*_ = exp (−*d*_*ij*_*/α*), where *d*_*ij*_ is the Euclidean distance between the ith and jth subjects, and *α* is a regularization factor). We evaluated their performance in terms of three different measures: modularity Q, reproducibility, and effectiveness of clinical interpretation. The reproducibility captures how a method provides consistent results over different datasets, which was measured by a fraction of consistently assigned subjects to each subtype on average. We divided the dataset into 10 subsets and repeated our method over them 10 times excluding one subset in turn. We then computed the average fraction over 10 runs. It is also important that resulting subtypes share a certain neuropsychological characteristics to be clinically useful.

### Inter-dataset consistency of subtyping

We tested how significantly each subtype obtained in the SMC dataset was matched with its corresponding subtype obtained in the ADNI validation dataset using permutation testing. We sought to determine whether the average similarity evaluated between the correctly matched subtypes (e.x. the MT subytpe in the SMC dataset and the MT subtype in the ADNI validation dataset) was higher than the other random matchings using permutation testing (see Supplementary materials for details).

### Cortical atrophy hallmarks of each subtype

We defined ‘*hallmarks’* of cortical atrophy for each subtype, which are the characteristic regions with severe atrophy. We first performed the ROI-based analysis of cortical thickness, where 34 cortical ROIs are pre-defined for each hemisphere^57^. We obtained normalized thickness values by dividing the average thickness of each ROI by the mean cortical thickness of the entire cortices. As our subtyping method clusters subjects based on the shape of cortical atrophy patterns rather than the level of overall cortical atrophy, a simple mean value of cortical thickness in a subtype does not represent the characteristics of the subtype. We compared the normalized cortical thickness of each ROI using permutation-based ANCOVA (see Supplementary material), controlling for age, gender and education level. We then selected hallmark regions that survived after the false discovery rate (FDR) procedure^58^,^59^ and revealed a distinct cortical atrophy pattern for each subtype.

### Statistical Analysis

Comparison of demographic data or standardized neuropsychological test scores between the three subtypes of AD was assessed using χ^2^ test or one-way analysis of variance (ANOVA), and a Least Significant Difference (LSD) test was conducted for post-hoc analysis. For comparison of neuropsychological test scores between the AD subtypes in the ADNI validation dataset, permutation-based analysis of covariance (ANCOVA) was used, adjusted for age, gender, and education (see Supplementary Material). All statistical operations for the demographic data and standardized neuropsychological test scores were performed using PASW Statistics 21 (SPSS, Chicago, IL) software except for permutation-based ANCOVA. Two-sided p-values less than 0.05 (*p* < *0.05*) were considered statistically significant. We employed random field theory^60^, and permutation-based ANCOVA for cortical atrophy comparisons (see Supplementary Material). All statistical operations and analyses of MR images were conducted using MatLab (Version 2014b, Mathworks, Natick, USA), SurfStat (RFT and visualization of cortical atrophy) and our in-house software (permutation testing).

### Data availability

We provided our in-house analysis software in our webpage (http://bia.korea.ac.kr/software/AD_subtyping/) along with the pre-processed test data set. Using the provided test data set, one can reproduce our results.

## Additional Information

**How to cite this article:** Park, J.-Y. *et al*. Robust Identification of Alzheimer’s Disease subtypes based on cortical atrophy patterns. *Sci. Rep.* **7**, 43270; doi: 10.1038/srep43270 (2017).

**Publisher's note:** Springer Nature remains neutral with regard to jurisdictional claims in published maps and institutional affiliations.

## Supplementary Material

Supplementary Information

## Figures and Tables

**Table 1:** Demographic and clinical characteristics of the study population.

	SMC dataset			ADNI validation dataset		
	CN (n = 320)	AD (n = 225)	P-value	CN (n = 158)	AD (n = 131)	P-value
Gender, female, n (%)	188 (58.8)	149 (66.2)	0.077	84 (53.2)	57 (43.5)	0.065
Age at MRI (years)	70.0 ± 7.9	70.4 ± 9.0	0.648	76.2 ± 5.4	74.1 ± 7.4	0.007
Education (years)	11.2 ± 5.5	9.5 ± 5.8	0.001	15.9 ± 2.9	15.0 ± 2.9	0.866
K-MMSE	27.56 ± 2.55	20.96 ± 3.70	<0.001	29.17 ± 0.98	23.42 ± 2.25	<0.001
CDR-SB	0	3.08 ± 0.84	—	0	3.15 ± 0.82	—
APOE ε4 carrier (%)*	—	99/179 (55.3)	—	45 (28.5)	83 (63.4)	<0.001
APOE ε2 carrier (%)*	—	5/179 (2.8)	—	23 (14.5)	4 (3.1)	<0.001
Intracranial volume (liter)	1.31 ± 0.21	1.34 ± 0.21	0.051	1.53 ± 0.16	1.55 ± 0.18	0.249
Mean cortical thickness (mm)	2.36 ± 0.08	2.27 ± 0.11	<0.001	2.13 ± 0.10	2.00 ± 0.13	<0.001

Abbreviations - AD = Alzheimer’s disease; MT subtype = medial temporal-predominant subtype; P subtype = parietal-predominant subtype; D subtype = diffuse atrophy subtype; K-MMSE = Korean Version of mini-mental state examination (scored out of 30); CDR = Clinical dementia rating; CDR-SB = CDR sum of boxes (scored out of 18). APOE = Apolipoprotein E.

^*^APOE genotyping was performed in 179 of 225 patients.

**Table 2:** Neuropsychological test scores of each AD subtypes SMC dataset.

SMC dataset	AD subtypes				Comparison between AD subtypes			
	MT subtype	P subtype	D subtype	Total	P-value^a^	MT vs. P	MT vs. D	P vs. D
	n = 82	n = 79	n = 64	n = 225				
Attention								
Digit-span forward	−0.25 ± 1.15	−0.36 ± 1.10	−0.24 ± 1.05	−0.29 ± 1.10	0.740	0.513	0.946	0.498
Digit-span backward	−0.49 ± 1.03	−1.15 ± 1.18	−0.73 ± 1.59	−0.79 ± 1.28	**0.004**	**0.001**	0.261	0.055
Language								
K-BNT	−2.04 ± 1.60	−1.91 ± 2.31	−1.19 ± 1.63	−1.75 ± 1.91	**0.019**	0.659	**0.008**	**0.026**
Visuospatial function								
RCFT copy, score	−0.65 ± 1.76	−4.53 ± 5.29	−0.73 ± 1.19	−2.05 ± 3.84	<**0.001**	<**0.001**	0.890	<**0.001**
RCFT copy, time	0.10 ± 1.12	−0.60 ± 1.59	0.46 ± 0.76	−0.05 ± 1.31	<**0.001**	<**0.001**	0.093	<**0.001**
Visual memory								
RCFT, immediate recall	−1.70 ± 0.92	−2.11 ± 0.69	−1.61 ± 1.08	−1.82 ± 0.92	**0.002**	**0.004**	0.561	**0.001**
RCFT, delayed recall	−1.78 ± 0.78	−2.20 ± 0.65	−1.72 ± 0.91	−1.91 ± 0.80	<**0.001**	**0.001**	0.636	<**0.001**
RCFT, recognition	−1.95 ± 2.08	−1.76 ± 1.53	−1.84 ± 2.04	−1.85 ± 1.88	0.817	0.527	0.734	0.803
Verbal memory								
SVLT, immediate recall	−1.24 ± 1.18	−1.67 ± 1.26	−1.21 ± 1.04	−1.38 ± 1.19	**0.027**	**0.020**	0.897	**0.021**
SVLT, delayed recall	−2.15 ± 1.36	−2.40 ± 0.97	−2.44 ± 1.65	−2.32 ± 1.33	0.349	0.240	0.195	0.845
SVLT, recognition	−1.67 ± 1.35	−2.3 ± 1.75	−1.66 ± 1.36	−1.89 ± 1.53	**0.012**	**0.008**	0.961	**0.013**
Frontal executive function								
COWAT, semantic-animals	−1.36 ± 0.99	−1.68 ± 1.11	−1.05 ± 0.95	−1.39 ± 1.05	**0.002**	**0.049**	0.077	<**0.001**
COWAT, semantic-supermarket	−0.98 ± 0.90	−1.53 ± 0.84	−1.10 ± 0.85	−1.21 ± 0.90	<**0.001**	<**0.001**	0.379	**0.004**
COWAT, phonemic with 3 letters	−0.64 ± 1.29	−0.75 ± 1.30	−0.31 ± 1.75	−0.60 ± 1.43	0.216	0.634	0.205	0.085
Stroop test, color reading	−1.23 ± 1.35	−3.31 ± 1.61	−1.27 ± 1.28	−2.01 ± 1.74	<**0.001**	<**0.001**	0.870	<**0.001**
Calculation^b^	9.66 ± 2.81	9.03 ± 2.88	9.15 ± 3.35	9.28 ± 2.99	0.053	0.016	0.210	0.217
Ideomotor praxis^b^	3.96 ± 1.33	3.44 ± 1.62	3.85 ± 1.25	3.74 ± 1.44	**0.004**	**0.001**	0.110	**0.071**

^a^One-way analysis of variance (ANOVA) followed by Fisher’s least significant difference (LSD) post hoc test was used for comparison of continuous variables except for the calculation test. P-values of post hoc tests are shown in bold where statistically significant.

^b^In tests where standard scores were not available, analysis of covariance (ANCOVA) followed by Fisher’s least significant difference (LSD) post hoc test was used for comparison among the AD dementia subtypes.

MT subtype = medial temporal-predominant subtype; P subtype = parietal-predominant subtype; D subtype = diffuse atrophy subtype. K-BNT = Korean version of Boston Naming Test; RCFT = Rey–Osterrieth complex figure test; SVLT = Seoul verbal learning test; COWAT = controlled oral word association test. Standard scores (z-scores) were used in comparison as age, sex, and education level in years were different among the AD dementia subtypes.

**Table 3:** Comparison of modularity and reproducibility for subtyping methods.

Dataset	Methods	Q	Reproducibility
SMC dataset	Hierarchical Clustering (Euclidian Distance)	—	83.42%
	Hierarchical Clustering (Correlation)	—	86.87%
	Louvain method (Euclidian Distance)	0.0110 (0.1891^a^)	—b
	Louvain method (Correlation)	0.2202	92.25%
ADNI validation dataset^c^	Hierarchical Clustering (Euclidian Distance)	—	72.03%
	Hierarchical Clustering (Correlation)	—	89.03%
	Louvain method (Euclidian Distance)	0.0235 (0.1665^a^)	—b
	Louvain method (Correlation)	0.2464	92.53%

^a^To compute the modularity value, similarity matrix is required but it also affects the modularity value Q. Thus, we computed the value using the same similarity matrix with our method in order to observe the effects of the modular organization only.

^b^The Louvain method with the Euclidian distance raised only two subtypes and thus it is unfair to compare its reproducibility with other methods.

^c^The ADNI validation dataset contained an unknown subtype and we excluded this type in the reproducibility analysis.
