# Supplementary material for: Robust Identification of Alzheimer’s Disease subtypes based on cortical atrophy patterns
Source: Sci Rep. 2017 Mar 9;7:43270. doi: 10.1038/srep43270 (PMC5343676; doi:10.1038/srep43270)
Supplement: Supplementary Information [file srep43270-s1.pdf]

Title: Robust Identification of Alzheimer's Disease subtypes based on cortical atrophy patterns

Authors: Jong-Yun Park[a,1], Han Kyu Na[b,1], Sungsoo Kim[b], Hyunwook Kim[b], Hee Jin Kim[c,d], Sang Won Seo[c,d], Duk L. Na[c,d], Cheol E. Han[a,e 2], Joon-Kyung Seong [a,2], for the Alzheimer′s Disease Neuroimaging Initiative

[a]School of Biomedical Engineering, Korea University, Seoul, Republic of Korea

[b]Yonsei University College of Medicine, Seoul, Republic of Korea

[c]Department of Neurology, Sungkyunkwan University of Medicine, Seoul, Republic of Korea

[d]Department of Neurology, Samsung Medical Center, Seoul, Republic of Korea

[e]Department of Electronics and Information Engineering, Korea University, Sejong, Republic of Korea

[1] These individuals contributed equally to this study

[2] These individuals contributed equally to this article as co-corresponding authors

Supplementary Results

In this section, we report subtyping results in the ADNI validation dataset in the same order with the SMC dataset. They showed the same trends overall.

AD subtyping based on distinct cortical atrophy patterns in the ADNI validation dataset. When the same subtyping strategies were applied to eligible participants in the ADNI validation dataset, four subtypes of AD were classified: three of them corresponded to the MT (*n*=44), P (*n*=41) and D (*n*=34) subtypes respectively, and the remaining subtype showed a minimal atrophy level (*n*=12, see Discussion). The cortical atophy patterns of the MT and P subtypes in the ADNI validation dataset showed the same trend with those of the SMC dataset(**Supplementary Figure S1**), although the ADNI validation dataset showed more severe and slightly more spreading of cortical thinning compared to entries in the SMC dataset. For the D subtype, not only the statistically significant regions but also the atrophy map showed more widespread atrophy over the whole brain when compared to the SMC dataset. It is important to note that the D subtype did not consist of 'leftovers' which failed classification into the MT or P subtypes, rather, these patients shared a similar atrophy pattern which was distinguishable from the others.

We next sought to confirm whether the subtypes we identified in the SMC dataset also existed in the ADNI validation dataset. In addition to a qualitative comparison of the atrophied regions (**Figure 2 and Supplementary Figure S1**), we also performed quantitative comparison tests based on the similarity of cortical atrophy. Each subtype obtained in the SMC dataset was matched with its corresponding subtype obtained in the ADNI validation dataset (MT subtype: p=0.001; P subtype: p=0.009; D subtype: p=0.003; through permutation testing, see Methods for details).

Neuropsychological performances in the ADNI validation dataset. The proposed subtypes in the ADNI validation dataset (**Supplementary Table S2**) also showed consistent association with the neuropsychological test results as was observed in the SMC dataset. The impairment in frontal executive function was greater in the P subtype compared to the others as revealed by the trail making test A (p=0.019), digit symbol substitution (p=0.017), and ADNI-EF (p=0.034). Moreover, the number cancellation test score results were the worst in the P subtype, though it failed to reach statistical significance (p=0.062). The additional results for demographic distribution per subtype can be found in **Supplementary Tables S1**.

Supplementary Discussion

Comparison of subtypes between the SMC dataset and ADNI vadlidation dataset. We observed the identical subtyping in the ADNI validation set. AD patients in the ADNI population were classified into four subtypes with three of them were consistent to the MT, P, and D subtypes in terms of both cortical atrophy pattern and cognitive profile. This consistency of the subtypes was also confirmed through the average similarity analysis and the in-group proportion (IGP) analysis. Considering the difference in race, age, gender distribution and even the tests performed to measure cognition, the similar trend, implies that the subtypes proposed by the present study truely exist and exhibit distinct clinical characteristics across the subtypes.

Characteristics of the subtypes. P subtype showed the worst clinical presentation throughout the overall cognitive domain including attention, language, frontal executive function, visuospatial function and visual memory. The other two subtypes (MT and D) exhibited milder phenotype compared to the P subtype and showed similar impairment in cognition, except for the language domain where the MT subtype showed worse performance in confrontation naming compared to the D subtype. Moreover, the proposed three subtypes revealed distinct atrophy; the precuneus, supramarginal, inferior and superior parietal cortices in the P subtype, and the entorhinal, parahippocampal cortices and the temporal pole in the MT subtype.

Neuropsychological characteristics in the ADNI validation dataset. The ADNI validation dataset also showed that very mild AD can be classified into multiple subtypes and that the P subtype tends to exhibit more severe impairment in executive function. Among the tests measuring frontal executive function, the P subtype patients were found to be impaired in tests related to visual scanning performance and processing speed, as well as the frontal executive function (TMT, Digit Symbol Substitution Test, and Number Cancellation Test) [1-5]. These results are consistent with parietal and frontal atrophy in the P subtype. However, the Clock Drawing Test, which is known to be most relevant for visuospatial/executive function [6], did not identify any difference across the subtypes. The disparity between the external validation population and the SMC dataset in the visuospatial function test results (RCFT Copy vs. Clock Drawing Test) can be explained by the different difficulty levels of the test [6,7]. In the ADNI validation dataset, the scores were near the maximum (i.e. 5 points) in all three subtypes, which in turn suggests that the Clock Drawing Test was a relatively easy task for the very mild AD patients and that it is unable to distinguish subtly different visuospatial abilities between the three subtypes. Considering the difference in race, age, gender distribution, and the tests performed to measure cognition, the similar trend in the

external validation study (though not exactly the same) implies that the subtypes proposed are valid and associated with distinct clinical characteristics across the subtypes.

γ (gamma) regularization parameter in modular organization extraction. We controlled the level of subtyping using γ regularization parameter [8], similarly to the hierarchical clustering method that chooses a cutoff value of similarity to control the number of clusters. With smaller value of the regularization parameter we can obtain less number of subtypes. In our experiments, we controlled it to obtain three subtypes based on the previous post-mortem study [9]. Since effects of the parameter depend on the number of subjects, the values were differently chosen for two datasets (the SMC dataset: 0.9, the ADNI validation dataset: 0.93). In the ADNI validation dataset, the unknown subtype existed consistently with variable γ values. This unknown group shows very low cortical atrophy level which is also distinguishable to the other types in the ADNI validation dataset (**Supplementary Figure S4**). Though we excluded this group in this study, further investigation of this group is also an intriguing issue.

Supplementary Method

Subject recruitment and MR image acquisition of the SMC dataset. In the SMC dataset, we recruited 225 AD patients and 320 age, gender and education level matched cognitively normal subjects (CN) at Samsumg Medical Center. Written informed consent for the study was obtained from all patients and was approved by the Institutional Review Board of Samsung Medical Center. From June 2006 through December 2010, a total of 711 patients who visited the Memory Disorders Clinic at the Samsung Medical Center were diagnosed as probable AD after undergoing neuropsychological tests, high-resolution 3.0-tesla T1-weighted MRI (Philips 3.0T Achieva) as well as detailed clinical interviews. Diagnosis of probable AD dementia was made by two fellowship trained behavioral neurologists (S.W. Seo and D.L. Na) using the criteria from National Institute of Neurological and Communicative Disorders and Stroke and the Alzheimer’s Disease and Related Disorders Association (NINCDS-ADRDA) [10]. Among those diagnosed as probable AD, we included the patients with Clinical Dementia Rating sum of boxes (CDR-SB)$\leq$4 only (i.e., very mild AD) [11,12] and those with Mini-Mental State Examination (MMSE) lower than 27 [13,14]. In order to minimize the effect of factors contributing to cortical atrophy other than Alzheimer etiology, we limited the participants to those with minimal white matter hyperintensities (WMH) (visual rating of the largest diameter of deep WMH$<$10mm; cap and band of periventricular WMH$<$10 mm) as proposed by Clinical Research for Dementia Of South Korea (CREDOS) [15,16].

In addition, in order to rule out other causes of dementia blood tests including a complete blood count, blood chemistry test, vitamin B12, folate levels, syphilis serology, and thyroid function test were conducted in all patients and also patients with current or past psychiatric illnesses or neurological disorder such as schizophrenia, epilepsy, or encephalitis were excluded. Furthermore, patients with other structural lesions on brain MRI such as hydrocephalus, tumor, territorial infarction or intracranial hemorrhage, whether supra- or infratentorial, were excluded. Moreover, patients proven to be familial AD of the autosomal-dominant inheritance type were excluded, leaving a total of 227 AD patients. Lastly, 34 out of 227 AD patients underwent amyloid positron emitting tomography (PET) imaging and two patients who turned out to be amyloid negative (2/34, 5.9%) were excluded from this study. As a result the final study population consisted of 225 very mild AD patients for this present study. Of the 225 patients, 86 patients (38.2%) had early onset AD (EOAD, onset age<65 years) and the remaining 139 patients (61.8%) had late onset AD (LOAD, onset age≥65 years)

As a control group, 320 cognitively normal individuals were recruited to serve as age- sex- and education level matched controls for the 225 AD patients for subtyping based on structural MRI. All the normal controls met the following criteria [17]: (a) no current or past history of neurologic or psychiatric disorders, (b) normal cognitive function as determined by neuropsychological tests, and (c) a normal activities of daily living as determined using the Seoul–Instrumental Activities of Daily Living test [18].

T1 weighted MRI data was recorded using the following imaging parameters: 1 mm sagittal slice thickness, over-contiguous slices with 50% overlap; no gap; repetition time (TR) of 9.9 ms; echo time (TE) of 4.6 ms; flip angle of 8°; and matrix size of 240×240 pixels, reconstructed to 480×480 over a 240 mm field-of-view.

Subjects and MR image acquisition of the ADNI validation dataset. The inclusion and exclusion criteria are described at http://www.adni-info.org/. Further subject selection strategies applied to the ADNI validation dataset was similar to the aforementioned criteria used in the SMC dataset. Among those diagnosed as probable AD dementia fulfilling the NINCDS-ADRDA criteria [10], we included the patients with CDR-SB≤4 (very mild AD) and those with MMSE<27 [11-14]. To exclude patients with cortical atrophy affected by non-AD pathology such as vascular factors, we further limited to patients with the volume of WMH<1.5mL. As a result, a total of 131 probable AD patients were included in this study. Moreover, 158 age, gender, and education level matched normal elderly were used as a control group.

T1-weighted MR images were obtained using a standardized 1.5 Tesla MRI protocol of the ADNI-1

study [19]. In brief, the magnetization-prepared rapid gradient echo sequence was used with the following characteristics: sagittal plane, repetition time/echo time/inversion time 2,400/3/1,000 ms, flip angle 8°, 24 cm field-of-view, 192 × 192 in-plane matrix, and 1.2-mm slice thickness.

Neuropsychological tests in the SMC dataset. The cognitive function of each participant in the SMC dataset was assessed using a standardized neuropsychological assessment tool, the Seoul Neuropsychological Screening Battery (SNSB) [20-22]. The SNSB includes tests designed to measure attention, language, praxis, visuoconstructive function, verbal and visual memory, and frontal executive function [20,21]. Among these tests, the following tests could be quantitatively scored and used to represent each of the following cognitive domains: digit span test (forward and backward) for assessing the attention and working memory; the Korean version of the Boston Naming Test (K-BNT) for the language domain [22]; the Rey–Osterrieth Complex Figure Test (RCFT) for visuospatial constructional ability (RCFT, copy score and time) and for visual memory function, including immediate and 20-minute delayed recall, and recognition); the Seoul Verbal Learning Test (SVLT), a standardized and Korean-elderly-population-optimized verbal memory test consisting of three learning-free recall trials of 12 words, a 20-minute delayed recall trial for these 12 items, and a yes or no delayed recognition test, for assessing the verbal memory function; the phonemic and semantic Controlled Oral Word Association Test (COWAT), and the Stroop Test (word reading and color font naming) for assessing frontal executive function. In comparing the aforementioned neuropsychological test results, we used standard scores (z-scores derived based on age- and education-adjusted norms)[20,21] because the age, sex, and education level were different among the AD dementia subtypes. . In assessing the parietal lobe function, the Calculation Test scored by the number of correct trials out of 12 trials of written calculations (three trials each for addition, subtraction, multiplication, and division) [20-22] and Ideomotor Praxis Test scored by the number of correct items in miming the tool use out of 5 items (hammer, driver, scissors, key, and knife) were used [20-22]. However, the standard scores were not available in these two tests. In addition to the SNSB, we also used the Korean version of the Mini-Mental Status Exam (K-MMSE) and CDR-SB.

Neuropsychological tests in the ADNI validation dataset. The ADNI neuropsychological assessment procedures have been previously described [23,24]. Modified Alzheimer's Disease Assessment Scale-cognitive subscale (ADAS-cog) [25,26], the most widely used standard cognitive measure in the AD population, Digit Span Test, BNT [27], Rey Auditory Verbal Learning Test (RAVLT) [28], Clock Drawing Test [6], Trail Making Test (TMT)

[1,5], Digit Symbol Substitution Test [29] and Category Fluency Test were used [24]. Modified ADAS-Cog 13-item scale [25] included a number cancellation task and a delayed free recall task in addition to 11 original ADAS-Cog items [26] assessing cognitive domain including memory, language, praxis, and orientation. Test performance was assessed for errors and the total score was 85 points and higher scores indicated greater cognitive impairment. Attention was measured by digit span forward and digit span backward, tests assessing the verbal attention and working memory, respectively. The language domain was assessed by confrontation naming ability via the 30-item version BNT [27]. Clock Drawing Test (a test assessing the ability to draw a clock, scored on symmetry of number placement, correctness of numbers, the presence of two hands, and hand placement) was used to evaluate the visuospatial function [6]. In assessing the memory function, Rey Auditory Verbal Learning Test (RAVLT) was used [28]. Executive function and processing speed was assessed by means of TMT, Digit Symbol Substitution Test, Category Fluency Test (animal and vegetables) and Number Cancellation Test. TMT composed of two parts. In part A, patients were asked to draw lines connecting an array of numbers in sequential order within the allotted time limit, while in part B, patients were presented an array of numbers and letters and were asked to connect lines alternating between numbers and letters in sequential order. TMT part A aimed to assess processing speed and visual scanning, while TMT part B aimed to executive function in addition to the processing speed and visual scanning. Wechsler Digit Symbol Substitution Test was used to assess the visuo-attentional psychomotor speed. Composite scores such as ADNI-EF and ADNI-Mem were also used [30,31]. ADNI-EF is a composite score developed by using a modern psychometric theory to incorporate including tests related to the executive function: DSST, Digit Span Backward, TMT A and B, Category Fluency, and Clock Drawing Test [30]. Similarly, ADNI-Mem is a composite memory score derived from Rey Auditory Verbal Learning Test, ADAS-Cog, MMSE, and Logical Memory data [31].

Statistical tests of cortical atrophy. We compare the cortical thickness data of subtype group with that of CN using a 2-sample *t*-test with random field theory [32] using SurfStat (http://www.math.mcgill.ca/keith/surfstat). We corrected for age, gender and education level in this analysis; those measures differed between subtypes (**Table 1 & Supplementary Table S1**). Since our subtyping methods clustered subjects based on the cortical atrophy pattern not the level of overall cortical thinning, the distribution of cortical atrophy of each vertice did not follow the normal distribution. Thus we visualized the median of cortical atrophy as a cortical atrophy map in **Figure 2 & Supplementary Figure S1 (lower row)**. We compared the normalized cortical thickness of hallmark regions between subtypes using permutation-based ANCOVA and the false discovery rate (FDR)

procedure [33,34], by controlling age, gender, and education level. The post-hoc pairwise comparison was also performed through permutation-based ANCOVA and the FDR procedure over 3 pairwise comparisons. All statistical operations and analyses of MR images were conducted using MatLab (version 2014b, Mathworks, Natick, USA), SurfStat (RFT and visualization of the cortical atrophy) and our in-house software (permutation testing).

Permutation-based ANCOVA. We employed permutation-based ANCOVA for three groups, controlling for the effects of age, gender and education years. Specifically, we re-populated the dataset *N-1* times using random re-assignment (permutation) of all subjects into one of three groups under the assumption of full exchangeability, keeping the number of subjects in each group, where *N* is the number of permutations. We then computed *F*-values for the original assignment and *N-1* permuted sets through a simple ANCOVA, which forms a null distribution of *F*-values. Finally, we estimated the significance level of group difference by a fraction of the occurrence whose *F*-values were not less than the *F*-value of the original assignment. We used 10,000 as *N*. We also performed the pairwise comparisons using the permutation based ANCOVA and the FDR procedure.

Permutation testing for similarity matrix. We used permutation testing for similarity matrix to assert how the current modular organization is significant. It is based on the fact that the average within-group similarity is larger than the average between-group similarity if the distinction between groups were clear enough [35]. Specifically, we re-populated the dataset *N-1* times using random re-assignment (permutation) of all subjects into one of groups, where *N* is the number of permutations. We used the average within-group similarity subtracted by average between-group similarity as a representative statistics. Thus we computed this value for the original assignment and *N-1* permuted sets, which forms a null distribution of the representative statistics. Then we estimated the significance level by a fraction of the occurrence whose representative statistics were not less than the representative statistics of the original assignment. We used 10,000 as *N*.

We also used this method to evaluate how the clustering was similar between datasets. We computed the similarity matrix between subjects in the SMC dataset and subjects in the ADNI validation dataset. Then we computed a representative statistics as the average similarity between subjects of a certain subtype in the SMC dataset and subjects of the same subtype in the ADNI validation dataset subtracted by average similarity of the others. Then we repeated the procedure above to estimate the significance level.

References

1 Bowie, C. R. & Harvey, P. D. Administration and interpretation of the Trail Making Test. *Nat Protoc* **1**, 2277-2281, doi:10.1038/nprot.2006.390 (2006).

2 Huang, H. C. & Wang, T. Y. Visualized representation of visual search patterns for a visuospatial attention test. *Behav Res Methods* **40**, 383-390 (2008).

3 Joy, S., Fein, D. & Kaplan, E. Decoding digit symbol: speed, memory, and visual scanning. *Assessment* **10**, 56-65 (2003).

4 Lafont, S. *et al.* The Wechsler Digit Symbol Substitution Test as the best indicator of the risk of impaired driving in Alzheimer disease and normal aging. *Dementia and geriatric cognitive disorders* **29**, 154-163, doi:10.1159/000264631 (2010).

5 Tombaugh, T. N. Trail Making Test A and B: normative data stratified by age and education. *Arch Clin Neuropsychol* **19**, 203-214, doi:10.1016/S0887-6177(03)00039-8 (2004).

6 van Hout, H. & Berkhout, S. Inter-rater reliability of the clock-drawing test. *Age Ageing* **28**, 327-328 (1999).

7 Shin, M. S., Park, S. Y., Park, S. R., Seol, S. H. & Kwon, J. S. Clinical and empirical applications of the Rey-Osterrieth Complex Figure Test. *Nat Protoc* **1**, 892-899, doi:10.1038/nprot.2006.115 (2006).

8 Blondel, V. D., Guillaume, J.-L., Lambiotte, R. & Lefebvre, E. Fast unfolding of communities in large networks. *Journal of Statistical Mechanics: Theory and Experiment*, 1000 (2008).

9 Murray, M. E. *et al.* Neuropathologically defined subtypes of Alzheimer's disease with distinct clinical characteristics: a retrospective study. *The Lancet. Neurology* **10**, 785-796, doi:10.1016/S1474-4422(11)70156-9 (2011).

10 McKhann, G. *et al.* Clinical diagnosis of Alzheimer's disease: report of the NINCDS-ADRDA Work Group under the auspices of Department of Health and Human Services Task Force on Alzheimer's Disease. *Neurology* **34**, 939-944 (1984).

11 O'Bryant, S. E. *et al.* Staging dementia using Clinical Dementia Rating Scale Sum of Boxes scores: a Texas Alzheimer's research consortium study. *Arch Neurol* **65**, 1091-1095, doi:10.1001/archneur.65.8.1091 (2008).

12 O'Bryant, S. E. *et al.* Validation of the new interpretive guidelines for the clinical dementia rating scale sum of boxes score in the national Alzheimer's coordinating center database. *Arch Neurol* **67**, 746-749, doi:10.1001/archneurol.2010.115 (2010).

13 Kukull, W. A. *et al.* The Mini-Mental State Examination score and the clinical diagnosis of dementia. *J Clin Epidemiol* **47**, 1061-1067 (1994).

14 Petersen, R. C. *et al.* Alzheimer's Disease Neuroimaging Initiative (ADNI): clinical characterization. *Neurology* **74**, 201-209, doi:10.1212/WNL.0b013e3181cb3e25 (2010).

15 Shim, Y. S. *et al.* Effects of medial temporal atrophy and white matter hyperintensities on the cognitive functions in patients with Alzheimer's disease. *Eur Neurol* **66**, 75-82, doi:10.1159/000329277 (2011).

16 Noh, Y. *et al.* A new classification system for ischemia using a combination of deep and periventricular white matter hyperintensities. *J Stroke Cerebrovasc Dis* **23**, 636-642, doi:10.1016/j.jstrokecerebrovasdis.2013.06.002 (2014).

17 Noh, Y. *et al.* Anatomical heterogeneity of Alzheimer disease: based on cortical thickness on MRIs. *Neurology* **83**, 1936-1944, doi:10.1212/WNL.0000000000001003 (2014).

18 Ku HM, K. J., Kwon EJ, Kim SH, Lee HS, Ko HJ, Jo S, Kim DK. A study on the reliability and validity of Seoul-Instrumental Activities of Daily Living (S-IADL). *J Korean Neuropsychiatr Assoc* **43**, 189-199 (2004).

19 Jack, C. R., Jr. *et al.* The Alzheimer's Disease Neuroimaging Initiative (ADNI): MRI methods. *Journal of magnetic resonance imaging : JMRI* **27**, 685-691, doi:10.1002/jmri.21049 (2008).

20 Kang, Y. W. & Na, D. L. *Seoul Neurophsychological Screening Battery: Professional Manual*. (Human Brain Research & Consulting Co., 2003).

21 Kang, Y. W., Chang, S. M. & Na, D. L. *Seoul Neurophsychological Screening Battery: Professional Manual*. 2 edn, (Human Brain Research & Consulting Co., 2012).

22 Kim, H. & Na, D. L. Normative data on the Korean version of the Boston Naming Test. *Journal of clinical and experimental neuropsychology* **21**, 127-133, doi:10.1076/jcen.21.1.127.942 (1999).

23 Mueller, S. G. *et al.* Ways toward an early diagnosis in Alzheimer's disease: the Alzheimer's Disease Neuroimaging Initiative (ADNI). *Alzheimers Dement* **1**, 55-66, doi:10.1016/j.jalz.2005.06.003 (2005).

24 Park, L. Q. *et al.* Confirmatory factor analysis of the ADNI Neuropsychological Battery. *Brain Imaging Behav* **6**, 528-539, doi:10.1007/s11682-012-9190-3 (2012).

25 Mohs, R. C. *et al.* Development of cognitive instruments for use in clinical trials of antidementia drugs: additions to the Alzheimer's Disease Assessment Scale that broaden its scope. The Alzheimer's Disease Cooperative Study. *Alzheimer Dis Assoc Disord* **11 Suppl 2**, S13-21 (1997).

26 Rosen, W. G., Mohs, R. C. & Davis, K. L. A new rating scale for Alzheimer's disease. *Am J Psychiatry* **141**, 1356-1364 (1984).

27 Mack, W. J., Freed, D. M., Williams, B. W. & Henderson, V. W. Boston Naming Test: shortened versions for use in Alzheimer's disease. *J Gerontol* **47**, P154-158 (1992).

28 Barzotti, T. *et al.* Correlation between cognitive impairment and the Rey auditory-verbal learning test in a population with Alzheimer disease. *Arch Gerontol Geriatr Suppl*, 57-62, doi:10.1016/j.archger.2004.04.010 (2004).

29 Lezak, M. D. *Neuropsychological Assessment*. 3 edn, (Oxford University Press, 1995).
30 Gibbons, L. E. *et al.* A composite score for executive functioning, validated in Alzheimer's Disease Neuroimaging Initiative (ADNI) participants with baseline mild cognitive impairment. *Brain Imaging Behav* **6**, 517-527, doi:10.1007/s11682-012-9176-1 (2012).
31 Crane, P. K. *et al.* Development and assessment of a composite score for memory in the Alzheimer's Disease Neuroimaging Initiative (ADNI). *Brain Imaging Behav* **6**, 502-516, doi:10.1007/s11682-012-9186-z (2012).
32 Poline, J. B., Worsley, K. J., Evans, A. C. & Friston, K. J. Combining spatial extent and peak intensity to test for activations in functional imaging. *NeuroImage* **5**, 83-96, doi:10.1006/nimg.1996.0248 (1997).
33 Benjamini, Y. & Hochberg, Y. Controlling the false discovery rate: a practical and powerful approach to multiple testing. *J R Stat Soc Series B Stat Methodol*, 289-300 (1995).
34 Genovese, C. R., Lazar, N. A. & Nichols, T. Thresholding of statistical maps in functional neuroimaging using the false discovery rate. *NeuroImage* **15**, 870-878 (2002).
35 Kropf, S., Heuer, H., Gruning, M. & Smalla, K. Significance test for comparing complex microbial community fingerprints using pairwise similarity measures. *Journal of microbiological methods* **57**, 187-195 (2004).

Supplementary Figures

Cortical Atrophy Patterns

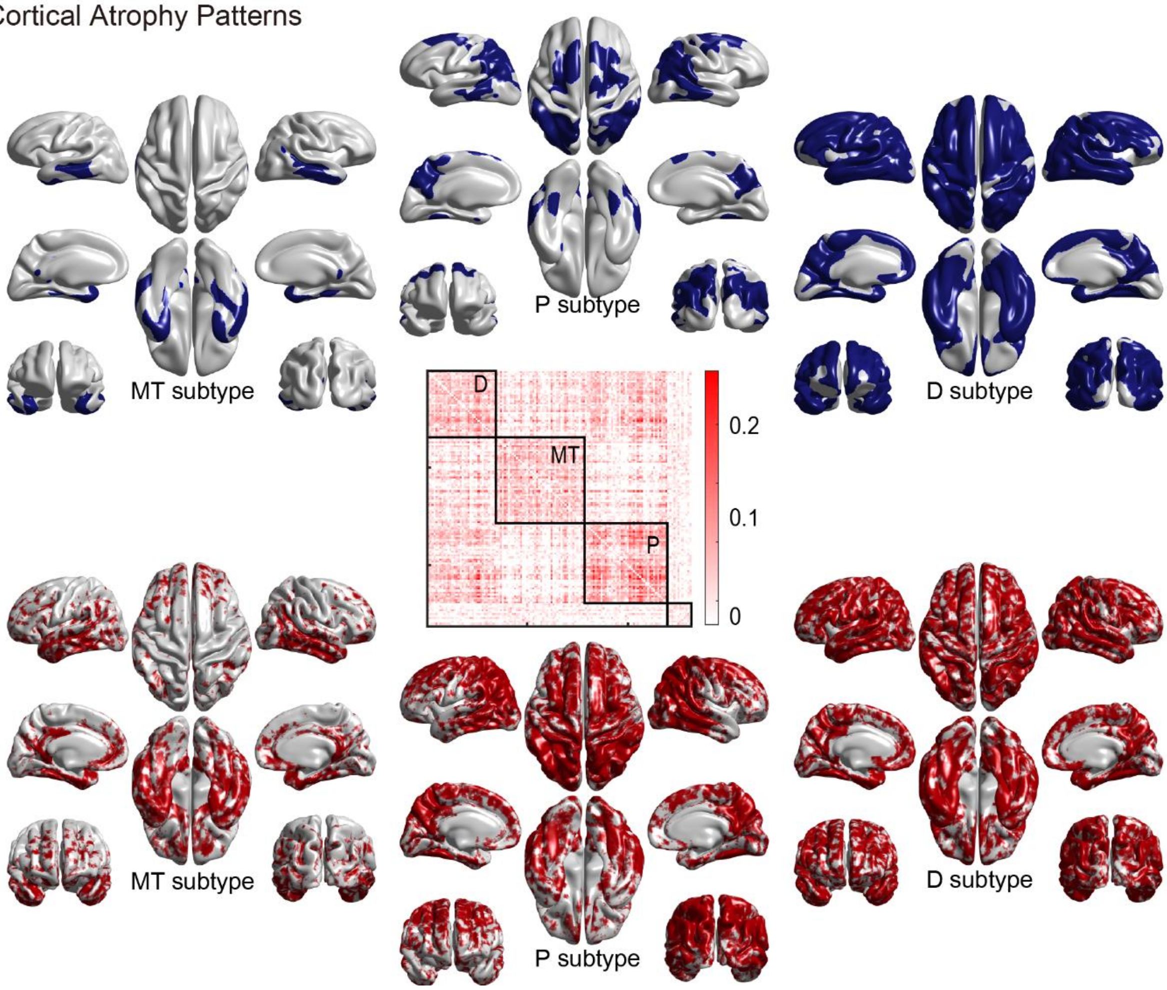

Supplementary Figure S1. **Cortical atrophy patterns for three AD subtypes using the ADNI validation dataset: MT (medial temporal-predominant), P (parietal-predominant), and D (diffuse) subtypes.** Modular organization of the subjects was achieved using defined similarity and reordered to illustrate subtyping where each square captures a subtype border. Group comparison results of cortical thicknesses between each subtype and CN was corrected using random field theory and regions with corrected $p<0.001$ are visualized ($p<0.05$ for the D subtype) with covariate age, gender and education. (upper row). Atrophy map shows medians of the cortical atrophy (z-scores) in each subtype ($-0.6 \leq z \leq -0.3$) (lower row).

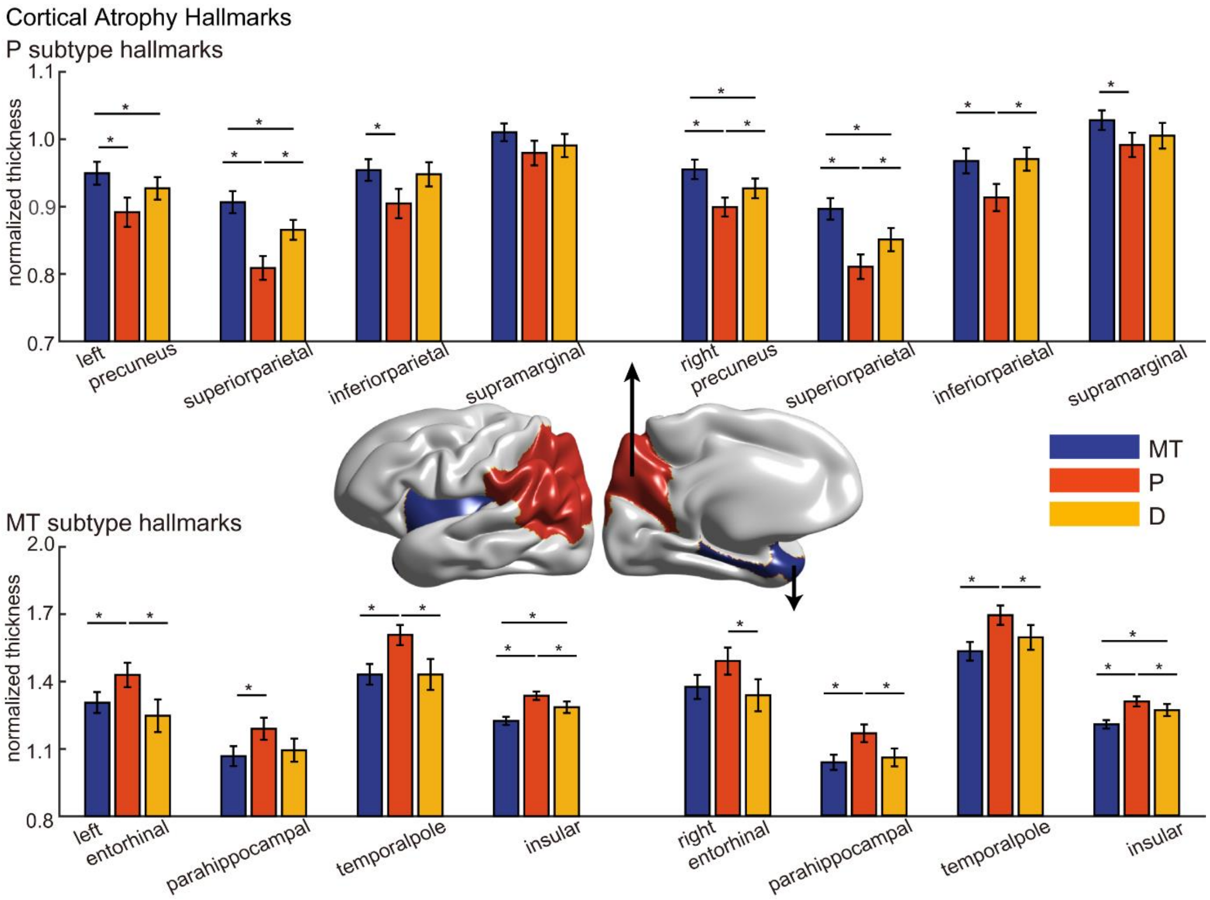

Supplementary Figure S2. Cortical atrophy hallmarks in each AD subtype in the ADNI validation dataset. Normalized cortical thicknesses of the subtype-specific hallmark regions are shown: P subtype hallmarks (upper right), MT subtype hallmarks (lower right) and D subtype hallmarks (left). Bar colors represent specific subtypes: blue (MT subtype), red (P subtype) and yellow (D subtype), where asterisks indicate statistical significance (permutation-based ANCOVA, FDR-adjusted)

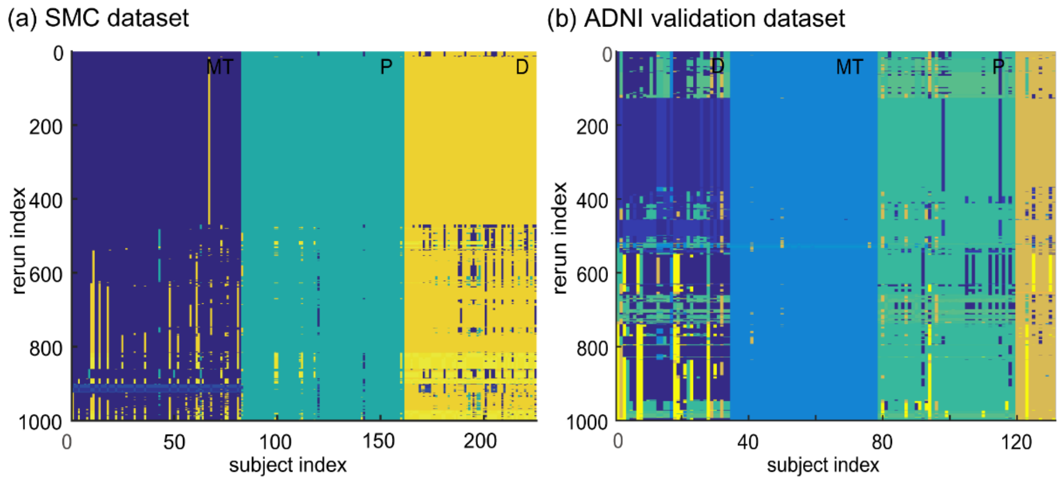

Supplementary Figure S3. Major voting results of Louvain Method for (a) the SMC dataset and (b) the ADNI validation dataset. The same color represents subjects of the same subtype.

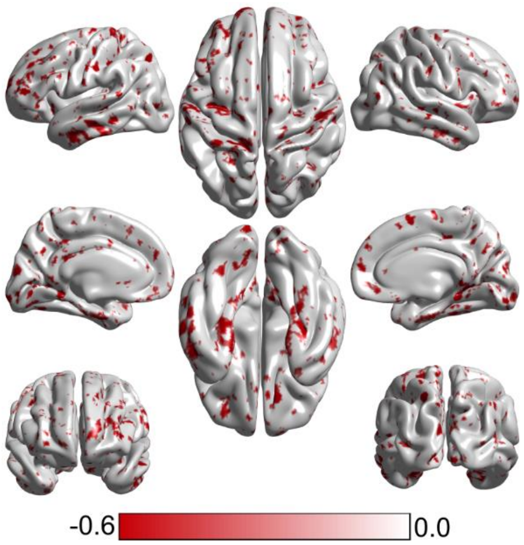

Supplementary Figure S4. Cortical atrophy patterns of the unknown subtype in the ADNI validation dataset. Atrophy map shows medians of the cortical atrophy (z-scores) in each group ($-0.6 \leq z \leq -0.3$). Group comparison results of the cortical thickness data between the subtype and CN showed no significant results after multiple comparison correction.

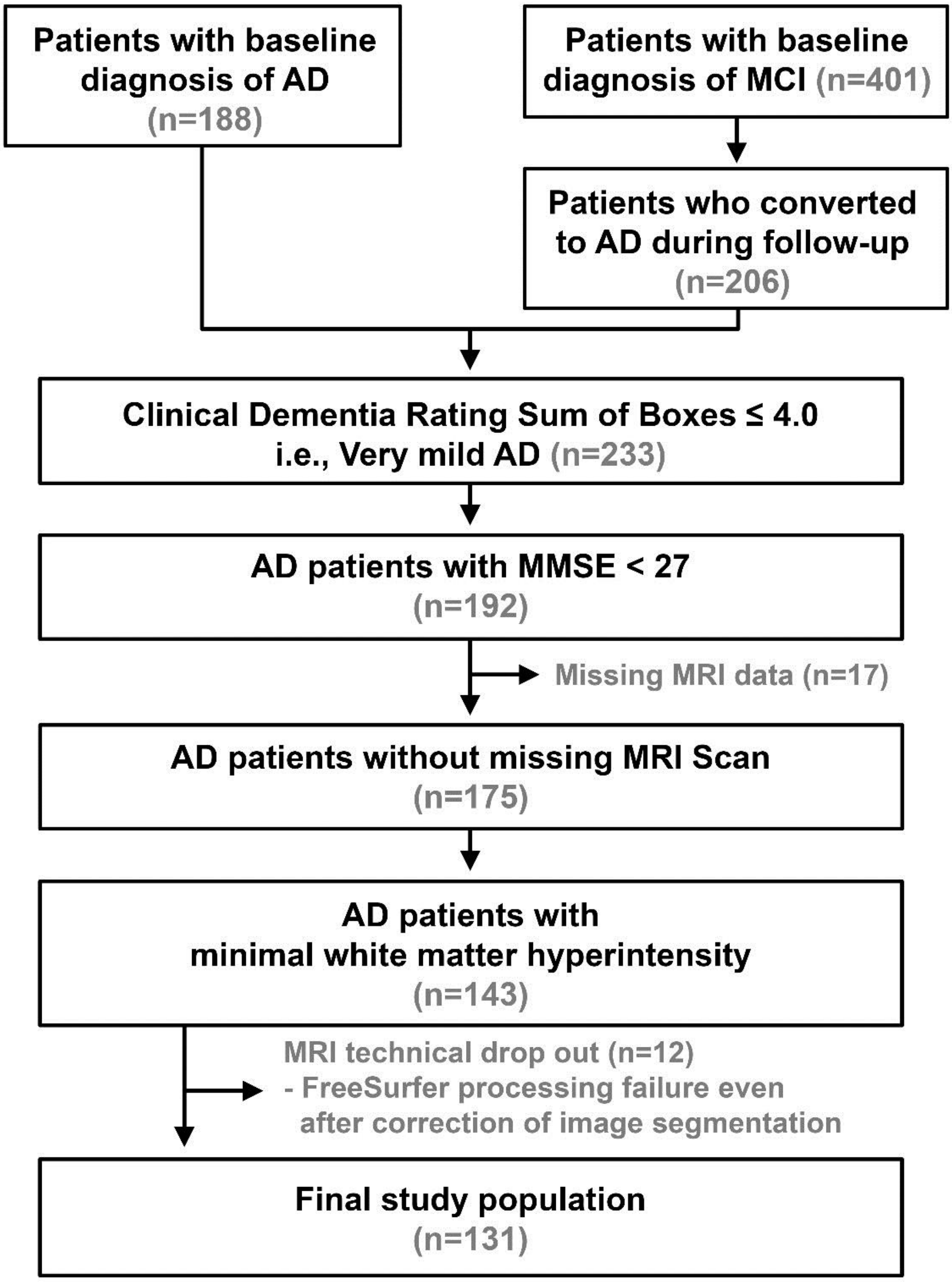

Supplementary Figure S5. Patient selection process in the ADNI validation dataset. The flow diagram shows the selection process of eligible patients from the ADNI-1 cohort.

Supplementary Table S1. Demographic and clinical characteristics of the study population in the SMC dataset and ADNI validation dataset

	Cognitively Normal subjects	AD dementia patients	P-value	Subtypes of AD on their early stage				P-value	MTvsP	MTvsD	PvsD
				MT subtype	P subtype	D subtype	Total				
**SMC dataset**	n=320	n=225		n=82	n=79	n=64	n=225				
Gender, female, n(%)	188 (58.8)	149 (66.2)	0.077	65 (79.3)	49 (62.0)	35 (54.7)	149 (66.2)	0.025	-	-	-
Age at MRI (years)	70.0±7.9	70.4±9.0	0.648	74.2±6.8	63.8±8.9	73.5±6.7	70.4±9.0	<0.001	<0.001	0.583	<0.001
Age at onset (years)	-	67.1±9.1	-	71.0±7.1	60.7±8.7	70.0±7.3	67.1±9.1	<0.001	<0.001	0.432	<0.001
Disease duration (months)	-	39.1±22.8	-	39.9±23.9	37.4±18.7	40.2±25.8	39.1±22.8	0.705	0.486	0.931	0.460
Education (years)	11.2±5.5	9.5±5.8	0.001	8.7±5.6	11.2±5.4	8.3±6	9.5±5.8	0.004	0.007	0.640	0.003
Diabetes mellitus	67 (20.9)	62 (27.6)	0.478	25 (30.5)	22(27.8)	15(23.4)	62 (27.6)	0.547	-	-	-
Hypertension	172 (53.8)	90 (40.0)	0.018	45 (54.9)	18(22.8)	27(42.2)	90 (40.0)	<0.001	-	-	-
Dyslipidemia	104 (32.5)	37 (16.4)	<0.001	15 (18.3)	10(12.7)	12(18.8)	37 (16.4)	0.398	-	-	-
Cardiovascular disease	34 (10.6)	21 (9.3)	0.605	10 (12.2)	4(5.1)	7(10.9)	21 (9.3)	0.300	-	-	-
K-MMSE	27.56±2.55	20.96±3.70	<0.001	20.87±4.05	20.92±3.30	21.13±3.75	20.96±3.70	0.911	0.921	0.676	0.748
CDR-SB	0	3.08±0.84	-	3.17±0.85	2.99±0.86	3.10±0.81	3.08±0.84	0.403	0.183	0.654	0.420
APOE ε2 carrier (%)*	-	5/179 (2.8)	-	2/63 (3.2)	1/65 (1.5)	2/51 (3.9)	5/179 (2.8)	0.603	-	-	-
APOE ε4 carrier (%)*	-	99/179 (55.3)	-	35/63 (55.6)	35/65 (53.8)	29/51 (56.9)	99/179(55.3)	0.403	-	-	-
Intracranial volume (liter)	1.31±0.21	1.34±0.21	0.051	1.31±0.17	1.35±0.24	1.36±0.21	1.34±0.21	0.352	0.284	0.176	0.730
Mean cortical thickness (mm)	2.36±0.08	2.27±0.11	<0.001	2.32±0.09	2.21±0.10	2.29±0.11	2.27±0.11	<0.001	<0.001	0.061	<0.001
**ADNI validation dataset**	n=158	n=131		n=44	n=41	n=34	n=119[a]				
Gender, female, n(%)	84 (53.2)	57 (43.5)	0.065	24 (54.5)	11 (26.8)	16 (47.1)	51 (42.8)	0.030	-	-	-
Age (years)	76.2±5.4	74.1±7.4	0.007	72.8±6.9	73.3±8.7	77.3±5.6	74.2±7.4	0.015	0.758	0.007	0.017
Education (years)	15.9±2.9	15.0±2.9	0.007	14.8±2.7	15.8±2.8	14.9±3.1	15.1±2.8	0.229	0.111	0.865	0.184
Diabetes mellitus	9 (5.7)	10 (7.6)	0.508	2 (4.5)	3 (7.3)	4 (11.8)	9 (7.6)	0.488	-	-	-
Hypertension	72 (45.6)	61 (46.6)	0.866	21 (47.7)	21 (51.2)	12 (35.3)	54 (45.4)	0.357	-	-	-
Dyslipidemia	62 (39.2)	60 (45.8)	0.261	20 (45.5)	17 (41.5)	17 (50.0)	54 (45.4)	0.761	-	-	-
Cardiovascular disease	27 (17.1)	28 (21.4)	0.356	8 (18.2)	9 (21.9)	9 (26.5)	26 (21.8)	0.680	-	-	-

MMSE	29.17±0.98	23.42±2.25	<0.001	23.25±2.05	23.37±2.47	23.38±2.24	23.33±2.24	0.959	0.813	0.798	0.975
Modified ADAS-Cog	9.43±4.32	26.38±6.98	<0.001	26.21±7.26	26.36±6.66	27.38±6.82	26.60±6.89	0.734	0.923	0.461	0.528
CDR-SB	0	3.15±0.82	-	3.24±0.77	3.09±0.89	3.29±0.65	3.20±0.78	0.478	0.368	0.756	0.252
APOE ε2 carrier (%)	23 (14.5)	4 (3.1)	<0.001	0 (0.0)	1 (2.4)	3 (8.8)	4 (3.4)	0.930	-	-	-
APOE ε4 carrier (%)	45 (28.5)	83 (63.4)	<0.001	29 (65.9)	25 (61.0)	24 (70.6)	78 (65.5)	0.682	-	-	-
Intracranial volume (liter)	1.53±0.16	1.55±0.18	0.249	1.55±0.19	1.60±0.18	1.50±0.17	1.55±0.18	0.064	0.230	0.210	0.019
Mean cortical thickness (mm)	2.13±0.10	2.00±0.13	<0.001	2.05±0.12	1.95±0.11	1.93±0.10	1.98±0.12	<0.001	<0.001	<0.001	0.338

Data are presented as mean ±SD for continuous variables and one-way analysis of variance (ANOVA) followed by Fisher's least significant difference (LSD) post hoc test was used for comparison of continuous variables. χ^2 and Fisher’s exact test was used for comparing the categorical variables.

Abbreviations - AD= Alzheimer’s disease; MT subtype = medial temporal-predominant subtype; P subtype = parietal-predominant subtype; D subtype = diffuse atrophy subtype; K-MMSE = Korean Version of mini-mental state examination (scored out of 30); CDR = Clinical dementia rating; CDR-SB = CDR sum of boxes (scored out of 18). APOE = Apolipoprotein E

* APOE genotyping was performed in 180 or 227 patients.

Supplementary Table S2. Neuropsychological test scores of each AD subtypes in the ADNI validation dataset

	Total	MT subtype (n=44)	P subtype (n=41)	D subtype (n=34)	P-value[a]	MTvsP	MTvsD	PvsD
**Attention**								
Digit Span Forward	7.58±2.01	7.25±1.66	7.83±2.14	7.71±2.25	0.523	0.267	0.478	0.732
Digit Span Backward	5.07±1.92	4.80±1.65	5.02±1.78	5.47±2.35	0.686	0.558	0.405	0.791
**Language**								
BNT, Spontaneous	22.66±5.92	22.57±6.34	23.49±5.88	21.79±5.40	0.918	0.889	0.774	0.684
**Visuospatial function**								
Clock Score	3.55±1.06	3.45±1.04	3.49±1.14	3.76±0.96	0.622	0.791	0.339	0.494
Clock Copy	4.41±0.79	4.48±0.70	4.29±0.90	4.47±0.75	0.425	0.207	0.792	0.360
**Memory**								
ADNI-MEM	-0.73±0.54	-0.77±0.59	-0.67±0.54	-0.77±0.47	0.685	0.440	>0.999	0.470
RAVLT, Immediate Recall	23.82±7.25	23.70±7.97	24.34±7.41	23.33±6.14	0.689	0.639	0.656	0.390
RAVLT, Learning	1.72±1.78	1.55±2.05	1.80±1.50	1.85±1.73	0.659	0.472	0.412	0.894
RAVLT, Delayed Recall	0.70±1.64	0.73±1.82	0.83±1.83	0.50±1.11	0.635	0.531	0.714	0.351
RAVLT, Recognition	7.78±3.87	8.27±3.48	7.59±4.26	7.38±3.91	0.492	0.629	0.235	0.480
**Executive function**								
ADNI-EF	-0.78±0.84	-0.75±0.87	-1.03±0.80	-0.53±0.79	**0.041**	0.082	0.371	**0.010**
Category Fluency - Animal	12.81±5.12	13.07±5.79	12.39±5.05	12.97±4.32	0.707	0.406	0.715	0.678
Category Fluency - Vegetable	8.14±3.44	8.52±3.79	7.41±3.30	8.53±3.05	0.487	0.273	0.959	0.335
Trail Making Test A	60.71±35.23	57.20±34.17	73.49±38.45	49.82±28	**0.019**	**0.043**	0.379	**0.007**
Trail Making Test B	194.12±90.29	194.07±85.84	211.1±90.75	174.21±93.75	0.191	0.233	0.480	0.075
Digit Symbol Substitution Test	28.4±12.47	30.14±13.66	24±10.88	31.47±11.45	**0.017**	**0.014**	0.810	**0.012**
Number Cancellation	18.42±8.09	19.95±8.21	15.85±8.33	19.53±7.02	0.071	0.030	0.790	0.084

[a]One-way analysis of variance (ANOVA) followed by Fisher's least significant difference (LSD) post hoc test was used for comparison of continuous variables. P-values of post hoc tests are shown in **bold** where statistically significant.

MT subtype = medial temporal-predominant subtype; P subtype = parietal-predominant subtype; D subtype = diffuse atrophy subtype

BNT = Boston Naming Test; RAVLT=Rey Auditory Verbal Learning Test,

ADNI-MEM = a composite score for memory in the Alzheimer's Disease Neuroimaging Initiative (ADNI), ADNI-EF = a composite score for memory in the ADNI

Supplementary Table S3. Neuropsychological test scores of EOAD and LOAD in SMC dataset

	Early onset Alzheimer's Disease (EOAD)								Late onset Alzheimer's Disease (LOAD)							
	Subtypes of AD				P value [a]				Subtypes of AD				P value [a]			
	MT (n=13)	P (n=55)	D (n=18)	Total (n=86)	Overall	MT vs. P	MT vs. D	P vs. D	MT (n=69)	P (n=24)	D (n=46)	Total (n=139)	Overall	MT vs. P	MT vs. D	P vs. D
**Attention**																
Digit-span forward	0.03±1.00	-0.45±1.02	-0.30±1.18	-0.35±1.05	0.327	0.141	0.398	0.583	-0.30±1.18	-0.16±1.25	-0.21±1.01	-0.25±1.13	0.838	0.588	0.685	0.841
Digit-span backward	-0.34±0.85	-1.35±1.22	-0.60±2.36	-1.05±1.50	**0.036**	**0.028**	0.634	0.074	-0.52±1.07	-0.69±0.92	-0.78±1.24	-0.63±1.10	0.459	0.520	0.224	0.744
**Language**																
K-BNT	-1.09±1.46	-2.08±2.60	-0.40±1.37	-1.57±2.33	**0.020**	0.157	0.405	**0.008**	-2.22±1.57	-1.53±1.47	-1.50±1.63	-1.86±1.60	**0.033**	0.065	**0.019**	0.953
**Visuospatial function**																
RCFT copy, score	-0.28±1.11	-5.65±5.77	-0.24±1.00	-3.75±5.34	**<0.001**	**<0.001**	0.979	**<0.001**	-0.72±1.85	-1.96±2.60	-0.92±1.21	-1.00±1.87	**0.018**	**0.005**	0.582	**0.026**
RCFT copy, time	0.60±0.92	-0.73±1.51	0.48±0.92	-0.29±1.45	**<0.001**	**0.002**	0.801	**0.002**	0.00±1.14	-0.30±1.78	0.46±0.70	0.10±1.19	**0.028**	0.284	**0.049**	**0.012**
**Visual memory**																
RCFT, immediate recall	-1.77±0.90	-2.18±0.49	-1.68±0.96	-2.02±0.70	**0.013**	**0.048**	0.725	**0.010**	-1.69±0.93	-1.96±1.01	-1.59±1.13	-1.70±1.01	0.354	0.266	0.611	0.153
RCFT, delayed recall	-2.01±1.02	-2.31±0.61	-2.06±1.02	-2.21±0.77	0.317	0.223	0.883	0.258	-1.73±0.72	-1.96±0.69	-1.60±0.84	-1.73±0.76	0.183	0.226	0.347	0.066
RCFT, recognition	-1.65±1.61	-1.68±1.29	-1.52±1.80	-1.64±1.43	0.930	0.960	0.805	0.705	-2.01±2.16	-1.95±2.00	-1.96±2.12	-1.98±2.11	0.989	0.914	0.897	0.997
**Verbal memory**																
SVLT, immediate recall	-1.31±1.26	-1.93±1.21	-0.93±0.99	-1.62±1.24	**0.005**	0.093	0.362	**0.002**	-1.22±1.17	-1.07±1.18	-1.33±1.04	-1.23±1.13	0.662	0.578	0.615	0.367
SVLT, delayed recall	-2.58±1.32	-2.57±0.82	-2.35±0.70	-2.53±0.88	0.640	0.963	0.469	0.363	-2.07±1.36	-2.01±1.17	-2.48±1.90	-2.19±1.53	0.306	0.856	0.165	0.221
SVLT, recognition	-2.17±1.53	-2.52±1.91	-1.92±1.51	-2.35±1.78	0.440	0.524	0.697	0.223	-1.57±1.30	-1.78±1.20	-1.56±1.31	-1.60±1.28	0.754	0.491	0.950	0.489
**Frontal executive function**																
COWAT, semantic-animal	-1.11±1.20	-1.85±1.16	-0.81±0.83	-1.53±1.18	**0.002**	**0.032**	0.470	**0.001**	-1.4±0.94	-1.28±0.90	-1.14±0.99	-1.30±0.95	0.356	0.568	0.153	0.581
COWAT, semantic-market	-0.83±1.01	-1.56±0.85	-1.20±0.94	-1.37±0.92	**0.025**	**0.010**	0.258	0.158	-1.00±0.88	-1.48±0.83	-1.07±0.82	-1.11±0.87	0.065	0.021	0.701	0.061
COWAT, phonemic	-0.52±1.44	-1.07±0.94	-0.68±0.82	-0.92±1.02	0.141	0.088	0.676	0.186	-0.66±1.27	0.01±1.68	-0.16±1.99	-0.38±1.62	0.146	0.090	0.136	0.685
Stroop test, color reading	-1.47±1.45	-3.82±1.46	-1.22±1.64	-2.97±1.89	**<0.001**	**<0.001**	0.673	**<0.001**	-1.18±1.33	-2.18±1.35	-1.29±1.14	-1.40±1.32	**0.006**	**0.002**	0.678	**0.010**
Calculation [b]	9.54±2.99	8.45±2.76	9.11±3.76	8.76±3.02	**0.014**	**0.006**	0.365	0.073	9.68±2.80	10.33±2.78	9.16±3.21	9.63±2.95	0.757	0.843	0.459	0.698
Ideomotor praxis [b]	4.00±1.41	3.25±1.66	4.06±1.20	3.53±1.57	**0.032**	**0.020**	0.674	0.061	3.95±1.33	3.88±1.48	3.77±1.27	3.88±1.33	0.152	0.860	0.140	0.597

Standard scores (z-scores) were used in comparison as age, sex, and education level in years were different among the AD dementia subtypes.

[a] One-way analysis of variance (ANOVA) followed by Fisher's least significant difference (LSD) post hoc test was used for comparison of continuous variables except for the calculation test. P-values of post hoc tests are shown in **bold** where statistically significant.

[b] In tests where standard scores were not available, analysis of covariance (ANCOVA) followed by Fisher's least significant difference (LSD) post hoc test was used for comparison among the AD dementia subtypes.

MT subtype = medial temporal-predominant subtype; P subtype = parietal-predominant subtype; D subtype = diffuse atrophy subtype

K-BNT = Korean version of Boston Naming Test; RCFT = Rey–Osterrieth complex figure test; SVLT= Seoul verbal learning test; COWAT = controlled oral word association test

Supplementary Table S4. Statistical analysis of subcortical volume in the SMC dataset and ADNI validation dataset

	Left hemisphere							Right hemisphere						
	Mean ± Standard deviation [a] (/10000)			Comparison between AD subtypes				Mean ± Standard deviation [a] (/10000)			Comparison between AD subtypes			
ROI names	MT subtype	P subtype	D subtype	P-value	MT vs. P	MT vs. D	P vs. D	MT subtype	P subtype	D subtype	P-value	MT vs. P	MT vs. D	P vs. D
**SMC dataset**	n=82	n=79	n=64					n=82	n=79	n=64				
Thalamus-Proper	45.91±7.72	49.08±8.58	46.46±8.03	0.09	0.226	0.626	0.226	44.38±7.21	47.26±8.50	44.87±7.23	0.173	0.426	0.471	0.299
**Caudate**	24.89±4.19	24.54±5.38	24.01±4.65	**0.008**	**0.035**	0.248	0.205	24.80±3.90	24.56±5.14	24.01±4.78	**0.03**	0.084	0.511	0.188
**Putamen**	33.99±6.28	34.32±8.17	33.64±6.40	**0.004**	**0.032**	0.903	**0.032**	33.15±6.60	33.32±7.32	32.79±6.57	**0.001**	**0.009**	0.871	**0.009**
**Pallidum**	11.69±2.43	12.04±2.44	11.07±2.26	**0.005**	**0.014**	0.14	0.14	9.91±2.00	10.49±2.07	9.76±1.87	0.119	0.239	0.899	0.239
**Hippocampus**	21.01±5.34	21.91±5.57	21.97±5.76	**0.009**	**0.028**	0.482	**0.028**	22.77±5.28	23.32±5.48	23.06±5.46	**0.014**	**0.04**	0.891	**0.04**
Amygdala	8.26±2.23	9.02±2.23	8.94±2.16	**0.024**	0.188	0.188	0.059	9.24±2.29	9.48±2.03	9.43±2.46	**0.039**	0.065	0.792	0.065
	n=44	n=41	n=34					n=44	n=41	n=34				
**ADNI validation dataset**														
**Thalamus-Proper**	38.31±4.54	40.34±5.04	38.98±3.63	**0.033**	**0.031**	0.216	0.379	39.00±3.89	40.97±4.89	38.60±3.08	**0.023**	0.052	0.897	0.065
Caudate	21.76±2.84	20.70±2.67	21.81±2.38	0.526	0.624	0.641	0.624	23.18±3.80	21.49±2.82	22.52±2.42	0.167	0.303	0.303	0.551
Putamen	28.91±3.72	29.43±3.58	28.86±3.40	0.352	0.313	0.964	0.313	28.67±3.57	28.53±4.17	27.77±3.42	0.218	0.553	0.302	0.256
Pallidum	10.14±1.25	10.34±1.15	9.82±1.20	0.278	0.316	0.701	0.316	9.28±1.07	9.55±1.05	9.05±0.97	0.349	0.348	0.69	0.348
**Hippocampus**	18.12±2.65	18.63±2.71	16.78±2.54	**0.019**	0.184	0.184	**0.017**	18.60±2.58	18.91±2.96	17.22±2.30	**0.037**	0.43	0.055	**0.038**
Amygdala	6.44±1.02	6.93±1.14	6.64±1.44	0.228	0.142	0.962	0.318	7.09±1.25	7.75±1.12	7.49±1.47	0.129	0.102	0.595	0.42

[a] Subcortical volume were normalized by dividing the ICV of each subject.

Supplementary Table S5. Statistical analysis of hallmarks in the SMC dataset and ADNI validation dataset

	Left hemisphere							Right hemisphere						
	Mean ± Standard deviation			Comparison between AD subtypes				Mean ± Standard deviation			Comparison between AD subtypes			
ROI names	MT subtype	P subtype	D subtype	P-value	MT vs. P	MT vs. D	P vs. D	MT subtype	P subtype	D subtype	P-value	MT vs. P	MT vs. D	P vs. D
**SMC dataset**	n=82	n=79	n=64					n=82	n=79	n=64				
**Banks of STS**	1.01±0.06	0.97±0.07	1.05±0.05	**0.000**	**0.037**	**0.001**	**0.000**	1.02±0.06	0.98±0.06	1.05±0.05	0.704	0.810	0.810	0.810
Caudal anterior cingulate	1.05±0.10	1.08±0.10	1.08±0.12	**0.033**	0.168	0.168	0.670	1.05±0.09	1.08±0.08	1.06±0.11	**0.000**	**0.016**	**0.000**	0.107
Caudal middle frontal	1.05±0.05	1.02±0.05	1.03±0.04	**0.016**	0.058	**0.015**	0.271	1.04±0.03	1.01±0.05	1.02±0.04	**0.004**	**0.007**	0.051	0.258
**Cuneus**	0.77±0.04	0.78±0.06	0.74±0.05	**0.001**	0.561	**0.001**	**0.004**	0.78±0.05	0.80±0.05	0.76±0.05	0.719	0.883	0.914	0.883
**Entorhinal**	1.11±0.16	1.23±0.15	1.19±0.15	**0.017**	**0.033**	**0.033**	0.947	1.25±0.18	1.37±0.19	1.32±0.17	**0.003**	**0.019**	0.219	**0.009**
Fusiform	1.06±0.05	1.07±0.07	1.10±0.05	**0.002**	0.480	**0.000**	**0.016**	1.04±0.05	1.05±0.07	1.07±0.05	**0.034**	**0.026**	0.420	0.091
**Inferior parietal**	1.01±0.03	0.93±0.06	0.99±0.03	**0.000**	**0.000**	0.071	**0.000**	1.00±0.03	0.94±0.05	1.00±0.04	**0.001**	**0.002**	0.246	0.057
Inferior temporal	1.08±0.05	1.07±0.07	1.12±0.05	**0.001**	0.917	**0.001**	**0.004**	1.09±0.06	1.09±0.06	1.12±0.06	**0.010**	**0.012**	**0.012**	0.668
Isthmus cingulate	0.90±0.07	0.87±0.07	0.90±0.08	0.083	0.127	0.900	0.437	0.83±0.07	0.82±0.07	0.83±0.07	**0.016**	0.055	**0.011**	0.430
Lateral occipital	0.87±0.04	0.85±0.06	0.87±0.05	**0.019**	0.123	0.992	**0.039**	0.89±0.04	0.87±0.05	0.89±0.04	0.247	0.966	0.200	0.255
**Lateral orbitofrontal**	1.06±0.04	1.12±0.07	1.11±0.04	**0.000**	**0.000**	**0.000**	0.282	1.03±0.05	1.08±0.06	1.08±0.05	**0.000**	**0.000**	**0.004**	0.088
Lingual	0.73±0.04	0.75±0.05	0.75±0.04	**0.024**	0.338	**0.005**	0.466	0.76±0.04	0.78±0.05	0.77±0.03	0.071	0.083	0.137	0.947
**Medial orbitofrontal**	0.93±0.06	1.00±0.07	0.98±0.06	**0.000**	**0.000**	**0.000**	0.538	1.00±0.06	1.05±0.06	1.03±0.06	**0.000**	**0.000**	**0.000**	0.121
Middle temporal	1.11±0.05	1.09±0.06	1.14±0.06	**0.000**	0.179	**0.004**	**0.004**	1.14±0.05	1.12±0.06	1.16±0.05	**0.046**	0.082	0.159	0.299
**Para hippocampal**	0.97±0.11	1.03±0.11	0.97±0.11	0.050	**0.013**	0.586	0.200	0.97±0.09	1.03±0.10	0.98±0.09	**0.006**	**0.003**	0.453	0.108
**Paracentral**	1.00±0.04	1.02±0.05	0.97±0.05	**0.000**	0.358	**0.000**	**0.000**	0.98±0.04	1.00±0.05	0.95±0.05	**0.000**	**0.000**	**0.000**	**0.023**
Parsopercularis	1.06±0.04	1.07±0.04	1.06±0.04	**0.016**	0.299	0.494	0.094	1.04±0.04	1.05±0.04	1.05±0.04	**0.000**	**0.000**	0.862	**0.001**
Parsorbitalis	1.07±0.06	1.11±0.09	1.09±0.06	**0.014**	0.066	0.066	0.381	1.05±0.06	1.07±0.07	1.06±0.06	**0.004**	**0.004**	0.082	0.619
Parstriangularis	1.01±0.04	1.04±0.05	1.02±0.04	**0.002**	**0.021**	**0.021**	0.144	1.00±0.04	1.02±0.05	1.01±0.04	**0.000**	**0.001**	0.478	**0.001**
**Pericalcarine**	0.66±0.05	0.69±0.05	0.64±0.05	**0.000**	**0.009**	**0.005**	**0.000**	0.64±0.04	0.67±0.06	0.62±0.05	**0.049**	**0.035**	0.493	**0.035**
Postcentral	0.87±0.04	0.87±0.04	0.85±0.04	**0.014**	0.970	**0.023**	**0.028**	0.86±0.03	0.88±0.04	0.85±0.04	**0.006**	**0.012**	**0.021**	0.706
Posterior cingulate	1.00±0.05	0.99±0.05	0.99±0.06	0.605	0.689	0.689	0.803	0.95±0.05	0.95±0.05	0.95±0.05	**0.005**	**0.023**	**0.003**	0.565
**Precentral**	1.04±0.04	1.06±0.04	1.01±0.04	**0.000**	0.180	**0.000**	**0.000**	1.04±0.04	1.07±0.04	1.00±0.05	**0.000**	**0.000**	**0.000**	0.640
**Precuneus**	0.97±0.03	0.92±0.05	0.95±0.04	**0.000**	**0.000**	**0.002**	**0.003**	0.96±0.03	0.92±0.05	0.94±0.03	**0.000**	**0.000**	**0.046**	0.051
Rostral anterior cingulate	1.06±0.08	1.10±0.07	1.08±0.08	**0.001**	**0.006**	0.162	0.060	1.05±0.07	1.12±0.09	1.09±0.08	**0.000**	**0.000**	**0.001**	0.106
Rostral middle frontal	0.96±0.03	0.96±0.04	0.96±0.03	0.896	0.907	0.907	0.907	0.97±0.03	0.97±0.04	0.97±0.04	0.786	0.661	0.661	0.661
**Superior frontal**	1.14±0.03	1.14±0.04	1.12±0.03	**0.024**	0.972	**0.022**	0.055	1.13±0.03	1.13±0.04	1.11±0.03	**0.002**	**0.000**	0.304	0.073
**Superior parietal**	0.95±0.04	0.90±0.05	0.92±0.03	**0.000**	**0.000**	**0.000**	0.075	0.96±0.03	0.91±0.04	0.92±0.03	**0.000**	**0.000**	**0.001**	**0.000**
**Superior temporal**	1.05±0.03	1.08±0.04	1.08±0.04	**0.000**	**0.004**	**0.000**	0.974	1.08±0.04	1.11±0.06	1.10±0.04	**0.000**	**0.000**	0.736	**0.000**
**Supra marginal**	1.02±0.03	0.99±0.04	1.02±0.03	**0.000**	**0.000**	0.248	**0.005**	1.03±0.03	1.01±0.04	1.01±0.03	**0.035**	0.084	0.100	0.574
Frontalpole	1.16±0.09	1.16±0.12	1.15±0.10	0.849	0.927	0.927	0.927	1.14±0.09	1.15±0.09	1.14±0.12	0.062	0.068	0.647	0.122
**Temporalpole**	1.38±0.15	1.50±0.12	1.48±0.12	**0.000**	**0.004**	**0.000**	0.870	1.43±0.17	1.55±0.16	1.51±0.12	**0.000**	**0.000**	0.827	**0.000**
Transverse temporal	0.93±0.06	0.96±0.07	0.92±0.07	0.076	0.259	0.541	0.138	0.95±0.06	1.00±0.07	0.94±0.07	0.218	0.345	0.345	0.345
**insular**	1.16±0.04	1.22±0.05	1.22±0.05	**0.000**	**0.000**	**0.000**	0.693	1.14±0.06	1.18±0.05	1.19±0.06	**0.000**	**0.000**	**0.002**	**0.002**

	n=44	n=41	n=34					n=44	n=41	n=34				
**ADNI validation dataset**														
Banks of STS	0.94±0.09	0.96±0.09	0.96±0.10	**0.000**	0.089	0.078	**0.000**	1.00±0.10	1.00±0.09	1.04±0.09	0.436	0.713	0.713	0.602
**Caudal anterior cingulate**	1.13±0.14	1.23±0.17	1.30±0.15	**0.007**	**0.001**	0.153	0.319	1.15±0.14	1.25±0.11	1.26±0.12	**0.001**	**0.005**	**0.005**	0.990
Caudal middle frontal	1.02±0.05	0.97±0.08	0.99±0.05	**0.001**	**0.005**	**0.005**	0.609	1.04±0.05	0.96±0.08	0.99±0.06	**0.000**	**0.000**	**0.002**	0.317
Cuneus	0.77±0.06	0.77±0.06	0.77±0.05	**0.016**	0.773	**0.033**	**0.033**	0.78±0.06	0.77±0.05	0.78±0.05	0.374	0.648	0.648	0.300
**Entorhinal**	1.31±0.16	1.43±0.18	1.25±0.22	**0.048**	0.052	0.081	0.505	1.37±0.18	1.49±0.20	1.34±0.21	**0.017**	0.064	0.214	**0.029**
Fusiform	1.06±0.08	1.10±0.08	1.07±0.08	**0.016**	0.646	**0.001**	0.137	1.05±0.05	1.11±0.06	1.07±0.09	**0.001**	**0.000**	0.385	0.062
**Inferior parietal**	0.95±0.05	0.90±0.07	0.95±0.05	**0.000**	**0.000**	0.252	**0.000**	0.97±0.06	0.91±0.07	0.97±0.05	**0.000**	**0.000**	0.722	**0.002**
**Inferior temporal**	1.10±0.08	1.16±0.09	1.16±0.06	**0.023**	0.118	**0.021**	0.204	1.13±0.06	1.20±0.06	1.19±0.09	**0.000**	**0.000**	**0.001**	0.670
Isthmus cingulate	0.94±0.07	0.97±0.08	1.00±0.07	0.858	0.769	0.769	0.769	0.90±0.08	0.94±0.09	0.96±0.09	**0.022**	0.066	**0.033**	0.967
Lateral occipital	0.87±0.05	0.87±0.06	0.88±0.05	**0.011**	**0.019**	0.578	**0.029**	0.89±0.05	0.89±0.07	0.90±0.06	0.187	0.862	0.168	0.168
**Lateral orbitofrontal**	1.10±0.07	1.18±0.07	1.14±0.07	**0.000**	**0.000**	**0.000**	0.679	1.10±0.05	1.18±0.07	1.16±0.06	**0.000**	**0.000**	**0.000**	0.083
Lingual	0.78±0.05	0.81±0.05	0.80±0.05	0.298	0.441	0.441	0.766	0.79±0.05	0.83±0.06	0.81±0.05	**0.006**	**0.003**	0.143	0.430
**Medial orbitofrontal**	1.01±0.06	1.10±0.08	1.07±0.07	**0.000**	**0.000**	**0.000**	0.179	1.00±0.08	1.11±0.09	1.06±0.08	**0.000**	**0.000**	**0.005**	**0.014**
**Middle temporal**	1.10±0.10	1.14±0.09	1.13±0.06	**0.004**	0.763	**0.002**	**0.013**	1.14±0.07	1.19±0.08	1.18±0.08	**0.014**	**0.009**	**0.028**	0.776
**Para hippocampal**	1.07±0.15	1.19±0.16	1.09±0.15	**0.007**	**0.003**	0.277	0.120	1.04±0.12	1.17±0.13	1.06±0.12	**0.000**	**0.000**	0.452	**0.004**
**Para central**	0.99±0.07	0.90±0.08	0.93±0.06	**0.000**	0.915	**0.000**	**0.001**	0.96±0.07	0.90±0.07	0.92±0.05	**0.000**	**0.000**	**0.024**	**0.043**
Parsopercularis	1.05±0.05	1.10±0.06	1.05±0.07	0.883	0.747	0.747	0.747	1.07±0.07	1.09±0.06	1.06±0.06	0.064	0.132	0.854	0.132
Parsorbitalis	1.12±0.10	1.18±0.08	1.16±0.11	0.117	0.208	0.261	0.261	1.13±0.09	1.17±0.07	1.15±0.10	0.074	0.146	0.248	0.248
Parstriangularis	1.00±0.05	1.06±0.08	0.99±0.06	0.532	0.704	0.704	0.704	0.99±0.05	1.03±0.07	0.99±0.07	**0.019**	**0.038**	0.615	0.108
Pericalcarine	0.64±0.06	0.62±0.04	0.63±0.04	**0.000**	**0.019**	**0.019**	**0.001**	0.63±0.05	0.63±0.04	0.65±0.05	0.163	0.883	0.207	0.207
Post central	0.83±0.05	0.80±0.05	0.79±0.04	**0.014**	0.451	0.053	0.053	0.84±0.05	0.80±0.04	0.80±0.04	**0.001**	**0.002**	**0.016**	0.345
**Posterior cingulate**	1.02±0.08	1.07±0.08	1.08±0.06	0.738	0.671	0.671	0.671	1.02±0.07	1.06±0.08	1.05±0.08	**0.029**	**0.042**	0.084	0.376
Pre central	1.01±0.05	0.95±0.06	0.94±0.05	**0.000**	**0.020**	**0.000**	**0.000**	1.00±0.05	0.92±0.07	0.93±0.06	**0.000**	**0.000**	**0.000**	0.193
**Precuneus**	0.95±0.06	0.89±0.07	0.93±0.05	**0.000**	**0.000**	**0.000**	**0.029**	0.96±0.05	0.90±0.05	0.93±0.04	**0.000**	**0.000**	**0.008**	**0.030**
**Rostral anterior cingulate**	1.12±0.10	1.25±0.12	1.22±0.13	**0.000**	**0.000**	**0.001**	**0.048**	1.15±0.11	1.27±0.11	1.27±0.13	**0.000**	**0.000**	**0.000**	0.947
Rostral middle frontal	0.95±0.04	0.96±0.06	0.96±0.05	0.594	0.741	0.756	0.741	0.96±0.05	0.97±0.06	0.96±0.05	0.951	0.880	0.880	0.880
Superior frontal	1.14±0.03	1.11±0.04	1.13±0.06	**0.011**	0.716	**0.025**	**0.033**	1.13±0.04	1.10±0.05	1.12±0.06	0.050	**0.017**	0.436	0.274
**Superior parietal**	0.91±0.06	0.81±0.06	0.87±0.04	**0.000**	**0.000**	**0.000**	0.905	0.90±0.05	0.81±0.06	0.85±0.05	**0.000**	**0.000**	**0.000**	**0.006**
Superior temporal	1.04±0.08	1.12±0.07	1.05±0.06	**0.036**	0.082	**0.026**	0.519	1.06±0.07	1.15±0.07	1.08±0.09	**0.000**	**0.000**	0.490	**0.008**
Supra marginal	1.01±0.04	0.98±0.06	0.99±0.05	**0.018**	0.116	0.059	0.229	1.03±0.05	0.99±0.06	1.01±0.06	**0.017**	**0.020**	0.064	0.760
Frontalpole	1.18±0.11	1.22±0.12	1.16±0.13	0.427	0.403	0.403	0.403	1.16±0.12	1.21±0.13	1.15±0.15	0.108	0.295	0.649	0.110
**Temporalpole**	1.43±0.16	1.61±0.15	1.43±0.21	**0.000**	**0.005**	**0.005**	0.537	1.53±0.14	1.69±0.14	1.59±0.16	**0.000**	**0.000**	0.100	**0.013**
Transverse temporal	0.91±0.09	0.92±0.09	0.87±0.10	**0.000**	**0.005**	0.185	**0.001**	0.97±0.09	0.96±0.13	0.90±0.12	**0.043**	0.851	**0.034**	0.062
**insular**	1.22±0.06	1.34±0.06	1.29±0.08	**0.000**	**0.000**	**0.000**	0.494	1.21±0.06	1.31±0.07	1.27±0.08	**0.000**	**0.000**	**0.002**	**0.008**

Supplementary Table S6. Comparison of clinical implications for subtyping methods in the SMC dataset

	Hierarchical Clustering (Euclidian Distance)	Hierarchical Clustering (Correlation)	Louvain method (Euclidian Distance) [a]	Louvain method (Correlation)
Attention				
Digit-span forward				
Digit-span backward	○[b]		○	○
Language				
K-BNT				●[c]
Visuospatial function				
RCFT copy, score	○		○	○
RCFT copy, time	○			○
Visual memory				
RCFT, immediate recall	○		○	○
RCFT, delayed recall	○		○	○
RCFT, recognition				
Verbal memory				
SVLT, immediate recall			○	○
SVLT, delayed recall				
SVLT, recognition				●
Frontal executive function				
COWAT, semantic fluency -animals	○		○	○
COWAT, semantic fluency -supermarket items	○	○	○	○
COWAT, phonemic fluency with 3 letters				
Stroop test, color reading	○		○	○

[a] Though the Louvain method with the Euclidian distance raised only two subtypes, we divided one of the group into two subtypes again using the same subtyping method, and the clinical implication was analyzed for these three subtypes.
[b] Significant results
[c] Significant results exclusively only in our method

Supplementary Table S7. Comparison of clinical implications for subtyping methods in the ADNI validation dataset

	Hierarchical Clustering (Euclidian Distance)	Hierarchical Clustering (Correlation)	Louvain method (EuclidianDistance)[a]	Louvain method (Correlation)
Attention				
Digit-span forward				
Digit-span backward				
Language				
BNT, Spontaneous		○ [b]		
Visuospatial function				
Clock Score		○		
Clock Copy		○		
Memory				
ADNI-MEM				
RAVLT, Immediate Recall				
RAVLT, Learning				
RAVLT, Delayed Recall		○		
RAVLT, Recognition				
Executive function				
ADNI-EF	○	○	○	●[c]
Category Fluency - Animal	○	○	○	
Category Fluency - Vegetable	○	○	○	
Trail Making Test A	○	○	○	●
Trail Making Test B	○	○	○	
Digit Symbol Substitution Test	○	○	○	●
Number Cancellation	○	○	○	

[a] Though the Louvain method with the Euclidian distance raised only two subtypes, we divided one of the group into three subtypes again using the same subtyping method. Similarly to the Louvain method with the correlation coefficients, we discarded the unknown subtype and the clinical implication was analyzed for this three subtypes.

[b,c] While our method showed that executive functions of the P subtype was most impaired (denoted as [c]), the results of the others asserted that the executive functions of the MT subtype is least impaired (denoted as [b]). Since all of the methods asserted [c] in the SMC dataset, methods except our method were not consistent across the population datasets.
